# Supplementary material for: Barriers and facilitators to facility-based delivery in rural Zambia: a qualitative study of women’s perceptions after implementation of an improved maternity waiting homes intervention
Source: BMJ Open. 2022 Jul 25;12(7):e058512. doi: 10.1136/bmjopen-2021-058512 (PMC9328096; doi:10.1136/bmjopen-2021-058512)
Supplement: Supplementary data [file bmjopen-2021-058512supp001.pdf]

SURVEY ID [ ]

Instrument ID:

The MAHMAZ Project
Endline Impact Evaluation – In Depth Interview Instrument **ENGLISH**

Target Audience:

Women who have delivered a child in the last 12 months, who are ≥ 15 years of age, and who live within the study facility catchment areas

Was verbal informed consent (or assent/consent if age 15-17) obtained for this interview during the process of informed consent for the household survey?

☐ **YES**

☐ **NO – STOP!** Thank the participant for their time. Do NOT proceed with the interview.

Step 1: *Read the following statement. Please repeat the statement translated into the local language based on primary languages.*

"Thank you for agreeing to participate in this interview. My name is ______. I will be asking you questions during this interview and recording your responses on this machine."

"We want to understand in greater detail your and your community's views on pregnancy and delivery and your perspectives on mothers' shelters. Please feel free to tell us only what you feel comfortable sharing. There are no right or wrong answers, so please be honest and help us to understand what is true for you and your community. Are you ready to begin?"

Step 2: *Proceed to the interview guide. Please probe to obtain as in-depth and specific information you can.*

.

Interviewer Name: ____________________

1. **Interview Date:**

				2	0	1	8

MM DD YY

2. **Time Start:**

[ ][ ] : [ ][ ]
H H M M

3. **Time Finish:**

[ ][ ] : [ ][ ]
H H M M

THIS FORM MUST BE TURNED INTO YOUR FIELD COORDINATOR FOR EVERY IDI YOU CONDUCT.

Fong RM, *et al. BMJ Open* 2022; 12:e058512. doi: 10.1136/bmjopen-2021-058512

SURVEY ID

READ THE DATE, UNIQUE ID, and START TIME ON THE RECORDING BEFORE YOU BEGIN THE INTERVIEW.

"Thank you for agreeing to speak with me today. I'm going to ask you some questions about people in your community and some questions about your own experience."

Theme 1: Delivery practices and location:

1a. Where do most pregnant women in your community deliver their babies? (e.g., home, health center, hospital) **Why do they do so?**

1b. What **factors** do people in your community mostly consider when deciding where to deliver?

Probe for (ask each separately after the woman has initially responded. We want more than just yes/no answers, ask for detail!):

- Do people consider **safety**? How?
- Do people consider **cultural preferences**? How?
- Do people consider **comfort**? How?
- Do people consider **cleanliness**? How?
- Do people consider **the opinions of others** – health worker, SMAG, spouses, mother-in-law, etc. How?
- Do people consider **distance**? How?
- Do people consider **transport**? How?
- Do people consider **cost of delivering at a facility**? How?
- Do people consider the **health facility requirements** for delivery? How?
- Do people consider the presence of a **Mothers' Shelter**? How?
- Do people consider the **quality of care provided** by health facility staff? How?
- Do people consider **local rules/laws**? How?

1c. Please tell me a story of **your delivery journey**, of all the places you went to or intended to go.

Rephrase if needed: What happened on the days leading up to delivery, the day you delivered, and in the days after?

Fong RM, *et al. BMJ Open* 2022; 12:e058512. doi: 10.1136/bmjopen-2021-058512

SURVEY ID [ ]

1d. Tell me what factors were involved in the decision of where you delivered your last baby? What was the most important of these?
Probe for (ask each separately after the woman has initially responded!):

- Do people consider **safety**? How?
- Do people consider **cultural preferences**? How?
- Do people consider **comfort**? How?
- Do people consider **cleanliness**? How?
- Do people consider **the opinions of others** – health worker, SMAG, spouses, mother-in-law, etc. How?
- Do people consider **distance**? How?
- Do people consider **transport**? How?
- Do people consider **cost of delivering at a facility**? How?
- Do people consider the **health facility requirements** for delivery? How?
- Do people consider the presence of a **Mothers' Shelter**? How?
- Do people consider the **quality of care provided** by health facility staff? How?
- Do people consider **local rules/laws**? How?

1e. Who helped you decide where to deliver your last baby? Of those, who was most influential for you when you were deciding where to deliver your baby? **Why?**
Probe for: *yourself, spouse, mother-in-law, aunties, self, SMAGs, health worker, etc.*

1f. Are there penalties for delivering at home? What are the penalties (monetary/non-monetary)? How much/how many? Who enforces them?

<u>Theme 2: Mother's Shelters</u>

2a. In general, what do people think of mothers' shelters in your community?

2a1. Do people in your community use mothers' shelters? **Why or why not?**

2b. Where do people in your community hear about mothers' shelters?
Probe with: *From men/spouses, mother-in-law, other mothers elder, family, chiefs, ANC, SMAG, health worker, radio etc.*

2c. In general, what do people in your community think of the quality of mother shelters?

SURVEY ID

Probe with:

- What do people say about their **comfort**?
- What do people say about their **safety**? **Why**?
- What do people say about how they are **managed**?
- What do people say about the **linkages with the health facility**?
- What do people say about the **classes offered** there?

2d. If you have stayed at the mothers' shelter, please tell me about **your overall experience**. Would you stay again? Why or why not?

2e. Please tell me a story about a **positive experience** you have had at or have heard about the mothers' shelter.

2f. Please tell me a story about a **negative experience** you have had at or have heard about the mothers' shelter.

2g. What makes it **hard to use** the mothers' shelter in your community?

2h. What makes it **easy to use** the mothers' shelter in your community?

2i. Who **owns** the mothers' shelter? Please explain.

2j. How can the mothers' shelter in your community be **improved**?

Theme 3: Preparedness and Costs

3a. What did you do to plan for your delivery?
Probe for: *costs, transport, supplies, baby clothes, etc.*

3b. About how far along were you in your pregnancy when you began to plan for delivery?

SURVEY ID

3c. Who helped you plan for delivery? What did they do to help?
Probe for: *health provider at ANC, SMAGs, mother-in-law, spouse, friends, etc.*

3d. You delivered your most recent child at (Health facility/hospital/home/other). **Tell me what it cost you to deliver your most recent baby?** Please be specific.

Probe for: *preparations, supplies, gifts, assistance fee, transport*

3e. Would the costs have been different if you delivered at (health facility/hospital/home/other)? If yes, how so?

- What is the largest cost associated with delivering a baby in the facility?
  - On average, how much does this cost?

3f. When you planned for delivery did you **save money**? If so, how did you save money?

3g. What were the main **challenges** associated with saving money for delivery?

3h. Do you think you **saved enough**? Please explain.

3i. Do other people in the community contribute to a woman's delivery savings?
Probe for: Money or non-monetary items

3j. Did you or someone you know take part in a **savings group**? Please tell me about that experience.

"I have finished with this in-depth interview. Is there anything you would like to add before we end?"

Thank you so much for your time.
