# Supplementary material for: Barriers and facilitators to facility-based delivery in rural Zambia: a qualitative study of women’s perceptions after implementation of an improved maternity waiting homes intervention
Source: BMJ Open. 2022 Jul 25;12(7):e058512. doi: 10.1136/bmjopen-2021-058512 (PMC9328096; doi:10.1136/bmjopen-2021-058512)
Supplement: Author's manuscript [file bmjopen-2021-058512.draft_revisions.pdf]

BMJ Open is committed to open peer review. As part of this commitment we make the peer review history of every article we publish publicly available.

When an article is published we post the peer reviewers' comments and the authors' responses online. We also post the versions of the paper that were used during peer review. These are the versions that the peer review comments apply to.

The versions of the paper that follow are the versions that were submitted during the peer review process. They are not the versions of record or the final published versions. They should not be cited or distributed as the published version of this manuscript.

BMJ Open is an open access journal and the full, final, typeset and author-corrected version of record of the manuscript is available on our site with no access controls, subscription charges or pay-per-view fees (http://bmjopen.bmj.com).

If you have any questions on BMJ Open's open peer review process please email info.bmjopen@bmj.com

BMJ Open

Barriers and facilitators to facility-based delivery in rural Zambia: A qualitative study of women’s perceptions after implementation of an improved Maternity Waiting Homes intervention

Journal:	*BMJ Open*
Manuscript ID	bmjopen-2021-058512
Article Type:	Original research
Date Submitted by the Author:	21-Oct-2021

Complete List of Authors:	Fong, Rachel; Boston University School of Public Health, Global Health<br>Kaiser, Jeanette; Boston University School of Public Health, Global Health<br>Ngoma, Thandiwe; Right to Care Zambia, Research<br>Vian, Taryn; University of San Francisco - School of Nursing and Health Professions<br>Bwalya, Misheck; mothers2mothers<br>Sakanga, Viviane; Amref Health Africa, Programmes<br>Lori, Jody; University of Michigan School of Nursing, Office for Global Affairs & PAHO/WHO Collaborating Center<br>Kuhfeldt, Kayla; Boston University School of Public Health, Global Health<br>Musonda, Gertrude; Africare Zambia<br>Munro-Kramer, Michelle; University of Michigan School of Nursing, Health Behavior & Biological Sciences<br>Rockers, Peter; Boston University School of Public Health, Department of Global Health<br>Hamer, D.; Boston University School of Public Health, Department of Global Health<br>Ahmed Mdluli, Eden; Project Hope<br>Biemba, Godfrey; National Health Research Authority, Pediatric Centre of Excellence<br>Scott, Nancy; Boston University School of Public Health, Global Health

Keywords:	QUALITATIVE RESEARCH, PUBLIC HEALTH, Health policy < HEALTH SERVICES ADMINISTRATION & MANAGEMENT

SCHOLARONE™
Manuscripts

I, the Submitting Author has the right to grant and does grant on behalf of all authors of the Work (as defined in the below author licence), an exclusive licence and/or a non-exclusive licence for contributions from authors who are: i) UK Crown employees; ii) where BMJ has agreed a CC-BY licence shall apply, and/or iii) in accordance with the terms applicable for US Federal Government officers or employees acting as part of their official duties; on a worldwide, perpetual, irrevocable, royalty-free basis to BMJ Publishing Group Ltd ("BMJ") its licensees and where the relevant Journal is co-owned by BMJ to the co-owners of the Journal, to publish the Work in this journal and any other BMJ products and to exploit all rights, as set out in our licence.

The Submitting Author accepts and understands that any supply made under these terms is made by BMJ to the Submitting Author unless you are acting as an employee on behalf of your employer or a postgraduate student of an affiliated institution which is paying any applicable article publishing charge ("APC") for Open Access articles. Where the Submitting Author wishes to make the Work available on an Open Access basis (and intends to pay the relevant APC), the terms of reuse of such Open Access shall be governed by a Creative Commons licence – details of these licences and which Creative Commons licence will apply to this Work are set out in our licence referred to above.

Other than as permitted in any relevant BMJ Author's Self Archiving Policies, I confirm this Work has not been accepted for publication elsewhere, is not being considered for publication elsewhere and does not duplicate material already published. I confirm all authors consent to publication of this Work and authorise the granting of this licence.

For peer review only

Barriers and facilitators to facility-based delivery in rural Zambia: A qualitative study of women's perceptions after implementation of an improved Maternity Waiting Homes intervention

Author Name	Highest Degree	Affiliation	Contact
Rachel M. Fong	MPH	Department of Global Health, Boston University School of Public Health, Boston, MA, USA	rmfong@bu.edu
Jeanette L. Kaiser	MPH	Department of Global Health, Boston University School of Public Health, Boston, MA, USA	jlkaiser@bu.edu
Thandiwe Ngoma	MPH	Department of Research, Right to Care Zambia, Lusaka, Zambia	thandiwe.ngoma@equiphealth.org
Taryn Vian	PhD	School of Nursing and Health Professions, University of San Francisco, San Francisco, CA, USA	tvian@usfca.edu
Misheck Bwalya	MSc	mothers2mothers, Lusaka, Zambia	bwalyamisheck@gmail.com
Viviane R Sakanga	MPH	Amref Health Africa in Zambia, Lusaka, Zambia	Vivianesakanga@gmail.com
Jody R. Lori	PhD	Office for Global Affairs & PAHO/WHO Collaborating Center, University of Michigan School of Nursing, Ann Arbor, MI USA	jrlori@umich.edu
Kayla J. Kuhfeldt	MPH	Department of Global Health, Boston University School of Public Health, Boston, MA, USA	kaylakuh@bu.edu
Gertrude Musonda	MPH	Africare Zambia, Lusaka, Zambia	gmusonda@africare.org
Michelle L. Munro-Kramer	PhD	Department of Health Behavior & Biological Sciences, University of Michigan School of Nursing, Ann Arbor, MI USA	mlmunro@umich.edu
Peter C. Rockers	ScD	Department of Global Health, Boston University School of Public Health, Boston, MA, USA	prockers@bu.edu
Davidson H. Hamer	MD	Department of Global Health, Boston University School of Public Health; Section of Infectious Diseases, Department of Medicine, Boston University School of Medicine, Boston, MA USA	dhamer@bu.edu
Eden Ahmed Mdluli	MPH	Project HOPE	edenamm@gmail.com

For peer review only - http://bmjopen.bmj.com/site/about/guidelines.xhtml

		7500 Old Georgetown Rd<br>Bethesda, MD 20814	
Godfrey Biemba	MSc.	National Health Research Authority, Pediatric Centre of Excellence, Lusaka, Zambia	gbiemba@gmail.com
Nancy A. Scott	DrPH	Department of Global Health, Boston University School of Public Health, Boston, MA, USA	nscott@bu.edu

Corresponding Author:

Rachel M Fong
801 Massachusetts Avenue
Crosstown Center, 3rd Floor
Boston, MA 02118 USA
Email: rmfong@bu.edu
Phone: 617-358-2184

For peer review only

For peer review only - http://bmjopen.bmj.com/site/about/guidelines.xhtml

ABSTRACT

Objectives: Women in sub-Saharan Africa face well-documented barriers to facility-based deliveries. An improved maternity waiting homes (MWH) model was implemented in rural Zambia to bring pregnant women closer to facilities for delivery. We qualitatively assessed whether MWHs changed perceived barriers to facility delivery among remote-living women.

Design: We administered in-depth interviews (IDIs) to a randomly-selected subsample of women in intervention (n=78) and control (n=80) groups who participated in the primary quasi-experimental evaluation of an improved MWH model. The IDIs explored perceptions and preferences of delivery location. We conducted content analysis and used the health belief model to interpret results.

Setting and participants: Participants lived in villages 10+ kilometers from the health facility and had delivered a baby in the previous 12 months.

Intervention: The improved MWH model was implemented at 20 rural health facilities.

Results: Over 96% of participants in the intervention arm and 90% in the control arm delivered their last baby at a health facility. Key barriers to facility delivery were distance and transportation, and costs associated with delivery. Facilitators included no user fees, penalties for home delivery, desire for safe delivery, and availability of MWHs. Most themes were similar between study arms. Both discussed the role MWHs have in improving access to facility-based delivery. Intervention arm participants expressed that the improved MWH model encourages use and helps overcome the distance barrier. Control arm participants either expressed a desire for an improved MWH model or did not consider it in their decision-making.

Conclusions: Even in areas with high facility-based delivery rates in rural Zambia, barriers to access persist. MWHs may be useful to address the distance challenge, but no single intervention is likely to address all barriers experienced by rural, low-resourced populations. MWHs should be considered in a broader systems approach to improving access in remote areas.

Trial Registration: ClinicalTrials.gov Identifier: NCT02620436

Key words: Mothers' shelter; Maternal health; Qualitative research; Community perceptions; Health behaviors

ARTICLE SUMMARY

Strengths and limitations of this study

1. The study was conducted after the implementation of a large-scale maternity waiting home intervention, which allows us to examine the differences in women's perceptions of barriers experienced between areas that received the Core Maternity Waiting Home Model and areas that did not receive the intervention.
2. We used a randomly selected, representative sample of the population, which is rare in qualitative research. The seven study districts were also culturally diverse and geographically dispersed. This method assured the best opportunity to capture a diversity of perceptions.

For peer review only - http://bmjopen.bmj.com/site/about/guidelines.xhtml

3. Our sample of women living in rural Zambia is a hard-to-reach population that likely face the greatest barriers to facility delivery due to their low socio-economic status, poor road infrastructure, limited transportation options, and low densities of facilities and skilled birth attendants.
4. The findings are subject to recall bias, as we interviewed women who delivered up to 12 months prior to data collection. However, topics of discussion surrounded social norm, which is less subject to recall bias than personal experiences and decision-making.
5. We conducted multiple rounds of data collection throughout the duration of the study, which likely resulted in increased awareness of maternity waiting homes among the control groups.

For peer review only

For peer review only - http://bmjopen.bmj.com/site/about/guidelines.xhtml

BACKGROUND

To reduce maternal and neonatal mortality, the World Health Organization (WHO) recommends that every birth occurs at an equipped health facility, attended by a skilled professional.[1] However, home births remain common in many areas, particularly in rural, low-resource settings. Individual and system level factors associated with births outside of equipped health facilities are well documented and include lack of access to facilities, long distances, lack of transportation, costs associated with delivery, and women's decision-making autonomy.[2–7]

The Government of the Republic of Zambia (GRZ) has sought to increase facility-based deliveries and reduce maternal mortality through an increasingly articulated policy landscape, monitoring of relevant indicators, and strategic partnerships to impact supply and demand of maternal health services. Within its National Health Strategic Plan (NHSP), which is published approximately every five years and sets out the country's health goals over that period, the GRZ has emphasized the importance of deliveries supervised by skilled health personnel. This has been included as a monitoring indicator since at least 2005, and subsequently as a main objective of the NHSP since at least 2010.[8,9] Additionally, in 2006, the GRZ eliminated user fees on primary health services, including maternal and child health services, first in rural areas then countrywide, to remove cost barriers and increase universal access to health services.[10]

The GRZ more clearly articulated its commitment to reducing maternal mortality and increasing maternal health more generally through its "Roadmap for accelerating the reduction of maternal, newborn, and child mortality," which mapped all stakeholder groups and organizations within the country and set out a multipronged, ambitious plan for achieving its goals.[11] Through the Saving Mothers, Giving Life (SMGL) initiative, formed in 2011, which combined GRZ efforts with multiple external funders and implementers, many of the plans set out in the roadmap were implemented. Between 2012 and 2016, SMGL significantly increased facility-based delivery rates from 63% to 90% and

For peer review only - http://bmjopen.bmj.com/site/about/guidelines.xhtml

reduced maternal mortality by approximately 40% in multiple districts of Zambia through intensive investments in increasing access to and improving quality of health services, care coordination, and community outreach programs.[12,13] These same results were not experienced in non-SMGL-supported districts.[12]

One strategy to increase facility-based deliveries is maternity waiting homes (MWHs), which in theory allow a pregnant woman to reside near a health facility in the weeks prior to her estimated delivery date.[7,14–16] With lower densities of equipped health facilities in rural areas, and poor road infrastructure, women residing in rural locations typically endure lengthy travel to deliver at a well-equipped health facility and often experience challenges accessing transportation.[17–19] In rural settings, the presence of quality MWHs has been associated with increased rates of facility-based delivery.[14,20,21]

Through formative research conducted in Zambia, the Maternity Homes Alliance (MHA), a partnership between the GRZ, two local non-governmental organizations (Right to Care Zambia and Africare), and two academic institutions (Boston University and the University of Michigan), designed a Core MWH Model targeting pregnant women living most remotely.[22,23] The MHA hypothesized that quality MWHs could remove distance barriers to facility delivery by allowing women to travel to facilities earlier than when they are in labor or when it becomes urgent. The MHA constructed 20 MWH following the Core MWH Model (referred to hereafter as the Core MWH Model) and evaluated the impact on facility delivery rates among women living more than 10 kilometers from their designated rural health facility.[24,25] In order to understand how the Core MWH Model influenced maternal perceptions of barriers and facilitators to facility-based delivery, we conducted a qualitative assessment among women living in villages 10+ kilometers from the health facility in catchment areas with the Core MWH Model compared to those living in control catchment areas without the Core MWH Model.

For peer review only - http://bmjopen.bmj.com/site/about/guidelines.xhtml

METHODS

Study setting

This study was conducted in seven rural districts in Zambia which received the SMGL intervention: Nyimba and Lundazi in Eastern Province; Choma, Kalomo and Pemba in Southern Province; and Mansa and Chembe in Luapula Province.[25] These districts are generally rural, with low population densities, and their main industries are agriculture.[26] The majority of residents, especially away from the few urban centers, are subsistence farmers who may make some additional income selling crops or doing piecework (short-term, casual work).[27]

The mixed methods baseline evaluation for this study found high facility delivery rates of 81.4% among women living more than 10 km from their rural health facility, with 15.3% of women still delivering at home and 3.2% delivering on the way to a health facility.[19] Qualitatively, these remote-living women discussed transport availability, distance, and cost of transport and supplies as continued barriers to facility-based deliveries. They also noted informal penalty fees being levied by traditional leaders against women who delivered at home, which had been previously reported in the literature.[19,28]

Intervention description

Described fully elsewhere, the MHA constructed 20 MWHs that met each of the three main pillars of the Core MWH Model: (1) Infrastructure, equipment and supplies to ensure a safe, comfortable, and functional structure; (2) Policies, management and finances to ensure local oversight and sustainability of the homes; and (3) Linkages and services to ensure integration with the formal health system.[25] The implementation of the Core MWH Model in all 20 sites was the same, with minor differences in how the partners and individual sites operationalized the policies, management, and finances pillar. The Core MWH Model was a multi-room structure with residential wards, latrines, a bathing area, a cooking space, and a veranda for socialization. All clinical care was provided at the health

For peer review only - http://bmjopen.bmj.com/site/about/guidelines.xhtml

facility, but health facility staff jointly oversaw the MWHs as part of its governing committee and checked in daily on waiting women. Education courses on maternal and child health topics were conducted weekly at each intervention site.

Rural health facilities implementing the standard of care for waiting women were selected as matched control sites.[25] The standard of care varied widely with some having no formal space for waiting women and others having a small, community-constructed MWH in generally poor condition.

Study design

Data for this analysis were collected from August to September 2018 as part of the endline data collection of the quasi-experimental, cluster-controlled impact evaluation.[25] A household survey was administered to a randomly selected cross-section of 2,330 participants, 1,217 in the intervention, 1,113 in the control sites who lived 10 or more kilometers from their designated rural health facility. A randomly selected subsample of participants (7%) also completed an in-depth interview (IDI) following the survey. The IDI guide had a series of open-ended questions with prompts to explore themes on personal and community perceptions of delivery practices and location. The questions included probes for various themes that arose during baseline data collection, including culture, cost, distance, and transportation, among others.[19]

Participants

A random sample of participants were asked to participate in the IDI following their household survey. Per the primary study eligibility criteria, all participants were at least 15 years of age or older and delivered a baby in the past 12 months, irrespective of location of delivery. To mitigate selection bias, we first randomly selected four villages per catchment area, then randomly selected one household in each village to participate in the IDI following the main survey. This helped to select a representative sample of women in the sample frame.[25]

For peer review only - http://bmjopen.bmj.com/site/about/guidelines.xhtml

Data collection

Data collectors fluent in English and the four relevant local languages were trained in human subjects' protection, electronic data collection using Android tablets, and qualitative research methods. Immediately following the quantitative household survey, selected participants were offered a short break before commencing the IDI, which took approximately 30 minutes to complete. All interviews were audio recorded with consent from the participant. Participant demographics, collected during the household survey, were linked to IDI participants through their unique study ID. Demographics were captured electronically on encrypted tablets using the SurveyCTO Collect software (Dobility, Inc, Cambridge, MA).[29]

Data management and analysis

All IDI recordings were translated and transcribed verbatim into Microsoft® Word. The transcripts were coded line by line in NVivo v12 (QSR International, Doncaster, Australia) by two research staff familiar with the context and study. [30,31] The main codes were created *a priori* using the established themes from the interview guide. Additional sub-codes were added during coding as themes emerged. The first few transcripts were coded by both research staff and a coding comparison was run in NVivo v12 (QSR International, Doncaster, Australia) using a kappa coefficient to mitigate researcher bias. Codes with poor agreement (kappa value <0.40) were discussed and agreed upon. Codes were reviewed throughout the coding process and merged if there was agreement that two or more codes contained similar content. Discrepancies were deliberated and resolved. Matrix queries in NVivo v12 (QSR International, Doncaster, Australia) were used during the analysis to organize and understand themes in the coded data. Themes with more than 10 participants (25% of the sample) were summarized and included in the results. Findings were triangulated within the context of the bigger impact evaluation. Quotations which included costs in Zambian Kwacha (K) were converted to US Dollars

For peer review only - http://bmjopen.bmj.com/site/about/guidelines.xhtml

(US$) using an approximate average exchange rate over the 12 months preceding data collection (K10 to US$1). Findings were reported using the Standards for Reporting Qualitative Research Checklist.[32]

Demographic data were cleaned and analyzed in SAS v9.4 (SAS Institute Inc., Cary, NC, USA). We present demographics for the recently delivered women and their households, and the women's primary outcomes of delivery at a health facility. We report the median and interquartile range (IQR) for non-normal data, means and standard deviations (SD) or proportions where appropriate.

Theoretical framework

Interpretation of the qualitative findings was guided by the Health Belief Model (HBM), which explains health behaviors as the presence or absence of multiple constructs, including susceptibility, seriousness, perceived benefits and barriers to a behavior, cues to action, and self-efficacy.[33] Based on summary results from a performance assessment of 46 HBM-guided investigations conducted by Janz and Becker, perceived barriers were the strongest indicator of behavior change.[33,34]

We assess remote, rural women's perceived barriers to and benefits of delivering at a health facility (the primary health behavior) among those living in catchment areas with and without the Core MWH Model. Barriers, such as the cost of delivering at a facility, act as hurdles for an individual. They impede them from engaging in the health-related behavior. We use the term "facilitator" when assessing perceived benefits. Facilitators help an individual decide to take action on a specific health behavior. An example might be the perception of facility-based deliveries as being safer.

Patient and Public Involvement

Key community members including women, men, elders and traditional leadership were consulted in the conceptualization and design of the Core MWH Model.[22,23] The final design also included input from stakeholders at all levels throughout the Ministry of Health. Through a process evaluation during implementation, end-users and the public were regularly consulted to understand the acceptability and sustainability of the intervention.[35–38]

For peer review only

For peer review only - http://bmjopen.bmj.com/site/about/guidelines.xhtml

Ethical considerations

Ethical approvals for this study were obtained from the Boston University Medical Campus Institutional Review Board (IRB; Protocol number H-34526) and the ERES Converge IRB in Zambia (Protocol number 2015-Dec-012). The Zambian Ministry of Health and National Health Research Authority granted approval. Additionally, the Provincial Health Officers, and the District Health Officer in each study district supported the study. Written informed consent and assent when necessary was obtained from participants prior to conducting the household survey and IDI. Trial Registration Number: NCT02620436

RESULTS

Participant Characteristics

Demographic characteristics for the 158 IDI participants were similar between the intervention and control groups. Participants in both arms lived a similar distance from their assigned health facility (12-13 km) and had about 6 years of education. More intervention participants (65.4%) reported having used a MWH than in the control arm (42.5%). More participants in the intervention arm delivered at a health facility (96.1%) compared to the control arm (90.0%).

Table 1. Qualitative participant characteristics for the endline observation of the MWH impact study, by study arm

	Intervention n = 78	Control n= 80
**Participant characteristics**		
**Woman's age in years**, median (IQR)	26 (21, 32)	29.5 (23, 35)
**Years of education**, mean (SD)	6.1 (3.2)	6.0 (3.4)
**Married/Cohabiting**, n (%)	68 (87.2)	71 (88.7)
**Gravida**, mean (SD)	4.0 (2.4)	4.5 (2.3)
**Parity,** mean (SD)	3.5 (2.4)	4.2 (2.3)

For peer review only - http://bmjopen.bmj.com/site/about/guidelines.xhtml

**Primigravida**, n (%)	16 (20.5)	10 (12.5)
**Four or more ANC visits**, n (%)	60 (75.0)	59 (75.6)
**Delivered at health facility or hospital**, n (%)	75 (96.1)	72 (90.0)
**Age of most recently delivered baby (months)**, mean (SD)	7.0 (3.7)	7.1 (3.6)
**District**, n (%)		
**Choma/Pemba**	15 (19.2)	11 (13.7)
**Kalomo**	16 (20.5)	21 (26.2)
**Nyimba**	8 (10.3)	8 (10.0)
**Lundazi**	20 (25.6)	20 (25.0)
**Mansa/Chembe**	19 (24.4)	20 (25.0)
**Household Characteristics**		
**Household size**, median (IQR)	6 (5, 8)	7 (5, 8)
**Dependency Ratio** [a], mean (SD)	1.5 (1, 2)	1.5 (1, 2)
**Travel distance to health facility (km)**, median (IQR)	12.1 (11, 15)	13.2 (11, 16)
**No electricity**, n (%)	77 (98.7)	79 (99.0)
**Unimproved sanitation** [b], n (%)	66 (84.6)	59 (73.7)

Abbreviations: IQR = interquartile range; SD = standard deviation; ANC = antenatal care; MWH = maternity waiting home

[a] Dependency Ratio = (children <16 years old + adults >65 years old)/adults > 16 years old.

[b] Unimproved sanitation: pit latrine without slab/open pit, bucket toilet, hanging toilet/latrine, no facility/bush/field.

For peer review only

Factors influencing delivery location

Two major barriers influencing facility-based delivery emerged: 1) distance and transportation, and 2) costs associated with delivery, while four key facilitators emerged: 1) no user fees, 2) penalties for home delivery, 3) desire for safe delivery, and 4) availability of MWHs (Table 2).

For peer review only - http://bmjopen.bmj.com/site/about/guidelines.xhtml

Table 2. Key barriers and facilitators to facility-based delivery reported by qualitative participants during the endline observation of the MWH impact study, by study arm

Key Themes	Intervention	Control
**Barriers influencing facility delivery**	**1. Distance & transportation**: health facility is far, have to prepare transport and money, buy fuel in advance or walk a long way; bad roads<br>**2. Costs associated with delivery**: need to prepare money for transportation (costs more if distance is farther) and buy required delivery supplies; need to prepare food and money for MWH stay	**1. Distance & transportation**: health facility is far and transportation is challenging, have to plan to walk early and wait at the facility or MWH if there is one or need to prepare transport and money<br>**2. Costs associated with delivery**: need to prepare money for transportation and buy required delivery supplies
**Facilitators influencing facility delivery**	**1. Costs associated with delivery**: no cost to deliver at a facility; no cost to stay at MWH<br>**2. Penalties for home delivery**: penalty fee for home delivery – pay the chief and health facility; penalty made women fear home delivery though some believed it is a hoax<br>**3. Safe delivery**: concerned about complications at home and afraid of dying; safer to deliver at facility, where staff can help with complications<br>**4. MWH availability**: good to move to MWH before delivery to avoid transportation difficulties because health facility is far; told by health facility staff to go to MWH at 8 months if you live far away; MWH is free and has amenities	**1. Costs associated with delivery**: no cost to deliver at a facility<br>**2. Penalties for home delivery**: penalty fees for home delivery – pay the chief and health facility; penalty made women fear home delivery<br>**3. Safe delivery**: concerned about delivery complications at home, and afraid of dying from complications; safer to deliver at facility, where staff can help with complications<br>**4. MWH availability**: women chose to go to health facilities with MWH no matter how far; good for those far away from the facility – can rest before delivery; no MWH but want one in their area; MWH availability does not matter, women will go where they can deliver safely

Distance and transportation

Participants in both study arms regarded distance and transportation as key barriers when deciding whether or not to deliver at a health facility. Participants generally acknowledged the health facility is far, so they have to prepare transportation (e.g. booking a car or taxi, borrowing a bicycle or ox cart) and put aside money in advance (Table 3, quotes a-d). No facilitators relating to distance and transportation arose in either the intervention or control groups.

For peer review only

Table 3. Quotations illustrating key themes as barriers or facilitators to facility-based delivery as reported by qualitative participants during the endline observation of the MWH impact study, by study arm

Key Themes	Theme as Barrier vs. Facilitator to Facility Delivery	Intervention	Control
**Distance and transportation**	**Barrier**	a) "Yes, that's the reason women go to the MWH early, transport is a major challenge." – Woman, Pemba District<br><br>b) "[The health facility] is quite far. We walked there. I started off around 3pm and reached there at 7pm." – Woman, Lundazi District	c) "The distance from here to the clinic is quite long and transport here is a big challenge, because you need to have money to pay." – Woman, Pemba District<br><br>d) "The distance [to the health facility] is very big and transport is a problem..." – Woman, Lundazi District
	**Facilitator**	No themes emerged	No themes emerged
**Costs associated with delivery**	**Barrier**	e) "I thought about where I was going to find money to get what is required. They tell you exactly what you need to buy when going for antenatal checkups so that you can prepare." – Woman, Lundazi District	f) "The basic costs that we face are buying baby clothes, delivery kit, transport, and buying food when you stay at the clinic as you wait to deliver." - Woman, Lundazi District<br><br>g) "This is why people deliver at home, because most have no money to prepare for delivery at facilities and when you get there without these [required items], nurses shout at us a lot." – Woman, Nyimba District
	**Facilitator**	h) "[The health facility] does not charge. They only ask us to prepare what to use during delivery and for our children too." – Woman, Chembe District	i) "That's why we prefer delivering from a health facility where it is free than in the village where you have to pay the village headman and other people." – Woman, Chembe District
**Penalties for home delivery**	**Barrier**	No themes emerged	No themes emerged
	**Facilitator**	j) "If you deliver at home, like me, when you go to the health facility to have the baby checked you will need to pay K200 (~US$20) before your baby is checked. The nurses are	k) **"**We have our traditional headmen who have partnered with the health facilities. If you deliver at home, you will pay a goat to the chief and then when you go to the health facility, they will make you pay K100 (~US$10) to get the

For peer review only - http://bmjopen.bmj.com/site/about/guidelines.xhtml

		the ones who give these penalties." – Woman, Kalomo District	growth monitoring card [for the baby]. Those are the local rules that we do follow." – Woman, Lundazi District
**Safe delivery**	**Barrier**	No themes emerged	No themes emerged
	**Facilitator**	l) "Different things [like complications, can] happen. For me, I went to the hospital and thought the baby will die. The doctor put me on a drip so that I could get better. They acted fast and I was written a letter and transferred to a bigger hospital." - Woman, Lundazi District	m) "They [health facility staff] will help you when a baby is in breach position. They will find ways of delivering a normal baby. But if you deliver from home and it [a breach delivery] happens, by the time you go to look for transport, you would have been dead." – Woman, Mansa District
**MWH availability**	**Barrier**	No themes emerged	No themes emerged
	**Facilitator**	n) "We mostly use ox-carts, but it is always better to go early and stay at the MWH until the day you deliver. I personally decided early that I needed to go to the MWH in case of difficulties with transport arrangements." – Woman, Kalomo District<br><br>o) "I considered the MWH. If I go there without pots I will be helped because they have pots there so I can cook before I deliver. Bed and blankets were also given." – Woman, Choma District	p) "Usually that [MWH] is the first thing we look at. Due to long distances to the health facility in this area, women choose to go deliver at the clinics where there is a MWH, no matter the distance. Second, women know that if you are staying at the MWH, the health facility staff will be checking you regularly to see if you and the baby are ok and that you can deliver without any problems." – Woman, Lundazi District

For peer review only

For peer review only - http://bmjopen.bmj.com/site/about/guidelines.xhtml

Costs associated with delivery

Delivery-associated costs were a frequently cited barrier. Participants reported that procuring supplies for their MWH stay posed an obstacle to a facility-based delivery (Table 3, quotes e and f). A few participants mentioned that one reason they still deliver at home is due to fear of getting embarrassed or shouted at by the health facility staff if they are unable to buy all the delivery requirements including bleach, a bucket, gloves, chitenge (traditional wrapping cloth), baby blanket, baby clothes, and nappies (Table 3, quote g). Similarly, a woman in the intervention group discussed how health facility staff ignore or give less attention to patients who had not procured the required supplies. However, participants acknowledged that the health facility does not charge for the delivery itself (Table 3, quote h) and there is no cost to stay at the MWH, which were frequently cited as a facilitator. Participants in both study arms perceived home delivery to cost more due to penalty fees, discussed further below (Table 3, quote i).

Penalties for home delivery

No barriers related to penalties for home delivery arose in either the intervention or control groups. Many participants discussed fearing home delivery because of the penalties as a facilitator, because they would have to pay either or both the health facility and to the chief (Table 3, quotes j and k). This facilitator emerged in both study arms and at all sites, though it was more salient for women in the control arm. Women reported payments to the chief in the form of a goat or chicken, and making monetary payments to the health facility staff (reported amounts between 5 – 25 U.S. Dollars, depending on the study site). Women believed the "law" came from either the government, the chief, or the health facility, and is enforced by the chief, health facility staff, and/or community health workers. When asked why the penalty exists, participants discussed preventing transmission of diseases such as HIV to the baby during delivery and preventing maternal mortality. A few participants also reported having to pay for their child's growth monitoring card at the health facility when they went for

For peer review only - http://bmjopen.bmj.com/site/about/guidelines.xhtml

postnatal care after delivering at home and considered this a “penalty” for their home delivery imposed by health facility staff. Attempts to avoid these penalties influenced women to delivery at health facilities.

Conversely, a few participants in the intervention arm believed the penalty fees for home delivery to be “a hoax” (a story that nurses pass around), because they had never seen it enforced in their communities. This belief appeared to diminish the facilitator effect of the penalties on increasing facility deliveries.

Safe delivery:

No barriers related to safe delivery arose in either the intervention or control groups. Despite the perceived barriers of distance, transportation, and cost, participants in both study arms regarded the health and well-being of the mother and baby as one of the top priorities for deciding where to deliver. Participants in both study arms emphasized the importance of delivering at a health facility as a facilitator, where trained health staff can help if any complications arose and expressed fear of dying from giving birth in their village (Table 3, quotes l and m). Many women gave examples of complications such as the positioning of the baby (breach) or losing blood, and they anticipated that the health facility had or would take care of them, give them blood or put them on “a drip.” Some participants added that if the health facility staff were not able to handle the complications, they would be able to refer women to a higher-level facility.

MWH Availability:

No barriers related to MWH availability arose in either the intervention or control groups. Participants in both study arms acknowledged that people in their communities consider the availability of an MWH when deciding where to deliver, especially if they live very remotely (Table 3, quote n). This was more frequently discussed as a facilitator by participants in the intervention arm, where women also noted the importance of the amenities provided at the MWH, such as beds, blankets, and cooking

For peer review only - http://bmjopen.bmj.com/site/about/guidelines.xhtml

utensils (Table 3, quote o). Although some participants in the control arm noted the availability of a MWH in their area, many wished for one of higher quality. Some participants even expressed their willingness to travel farther to a health facility with a MWH (Table 3, quote p). However, some participants in the control arm reported they do not consider a MWH when deciding where to deliver; rather they are willing to travel to wherever they can deliver safely regardless of whether a MWH is present.

DISCUSSION

This qualitative study explored differences in the factors remote-living women perceive as influencing the location of delivery in communities randomly assigned to receive the Core MWH model intervention and in communities randomly assigned to standard of care. We found that key facilitators influencing delivery location across both study arms included the perception of safe delivery, no user fees, MWH availability, and penalties for home delivery. These key findings support other literature emerging from the same areas in Zambia.[19,22,23,39]

Despite implementation of the Core MWH Model and other demand-generating interventions including SMGL, women in both arms still perceived barriers to facility-based delivery, including distance and transportation, and costs associated with delivery, consistent with baseline evaluation findings.[19] Health facilities in these rural districts serve catchment areas of 5,000 to 11,000 individuals.[40] Because of the geography and low population density in the rural areas, health facilities are spread out and those fully equipped to manage obstetric complications are even fewer. In the near future, human resource and cost requirements make it unlikely that the GRZ could feasibly bring health services, particularly basic emergency obstetric services, within 5km of all populations (a WHO core health service access indicator for health systems).[41] MWHs are an alternative option to make health facilities physically accessible to the most rural populations by allowing women to wait and removing the urgency and

For peer review only - http://bmjopen.bmj.com/site/about/guidelines.xhtml

stress around traveling long distances once they have gone into labor. Though MWHs address the distance barrier, they alone do not address the transportation barriers that persist here and that have been widely reported in areas similar to rural Zambia.[4,42,43]

Not surprisingly, MWH availability was more heavily discussed amongst participants in the intervention arm where the Core MWH Model was implemented, though the theme also emerged amongst control participants, indicating an overall awareness of MWH in the study areas. MWHs help women to overcome the distance and transportation barriers, allowing them to better plan and prepare to be close to health facilities when nearing delivery. Additionally, the proximity of MWHs to health facilities theoretically allows women to be easily attended by staff during the final weeks of pregnancy and when labor begins, increasing the likelihood for a safe delivery, one of the three perceived major drivers affecting women's decision to deliver at health facilities.

Women also explained that penalties for home deliveries influenced their decision making, saying they would deliver at a health facility to avoid paying penalties to the health facility or to traditional leadership, consistent with findings from two previous studies in rural Zambia.[28,44] However, some women noted they did not witness any penalties being enforced in their communities. Similarly, when rural Zambian women were previously asked to enumerate all costs associated with delivery, penalties among women delivering at home were insignificant compared to all other delivery costs.[45] Penalties may exist only as a rumor within these populations, or are levied in limited geographic areas. Further informal queries revealed that levying penalties for home delivery is not a law or official GRZ regulation, and that the government discourages such practices. Regardless, the belief that penalties for home delivery exist is a motivator for women to deliver at a facility.

The concern over the cost of facility-based delivery also persisted. Zambian women are required by facility staff to provide delivery supplies, though they perceive the need to provide new items, particularly baby clothes, when delivering at a health facility to avoid embarrassment or being shouted

For peer review only - http://bmjopen.bmj.com/site/about/guidelines.xhtml

at by staff.[39] A study in rural Zambia also found a strong negative association between women saving enough during pregnancy and feeling disrespected by facility staff. Women who had saved more before coming to a health facility for delivery were less likely to report feeling disrespected. [46] Previous research in these areas of rural Zambia found delivery supplies, such as clamps and cotton wool, represent a small percentage of the overall delivery expenditure, while baby clothes and a baby blanket were the drivers of expenses.[45] Consistent with our qualitative findings, this quantitative study also showed that expenditure differed by delivery location, with women delivering at facilities reporting higher expenditures than those delivering at home.[45]

Women perceived MWHs may reduce access barriers due to distance, facilitating increased use of delivery and postnatal health services among remote-living women corroborating the quantitative findings from this study.[24] However, perceived challenges with transportation and costs associated with delivering at a facility or utilizing a MWH pose a potential barrier to their use, consistent with other studies.[39,47] Interventions that effectively address these challenges should continue to be explored and implemented.

Study Strengths

The results of this study support barriers and facilitators to facility delivery already well established in the literature, including those found within this study population at baseline.[4,42,43] However, this study provides three unique contributions. First, it was conducted after the implementation of a large scale MWH intervention, allowing us to examine the differences in perceptions between barriers experienced in areas that received the Core MWH Model and in areas that did not receive it. Second, our method of using a randomly selected, representative sample of the population assured the best opportunity for diversity in perceptions. Qualitative samples are rarely a randomly selected, representative sample of a population. We were able to conduct largescale qualitative research among a random, more representative sample of this population. Lastly, the rural population sampled for this

For peer review only - http://bmjopen.bmj.com/site/about/guidelines.xhtml

study is a hard-to-reach population that likely faces the greatest barriers to facility delivery due to their low socio-economic status, poor road infrastructure, limited transportation options, and low densities of facilities and skilled birth attendants. Using the qualitative data collected through this strategy, we were able to more deeply understand what influences these women's delivery location across seven culturally diverse and geographically dispersed study districts, providing important information for policy makers and program implementers interested in serving these vulnerable populations.

Study Limitations

While this study has multiple strengths, there were also several limitations. First, our findings are subject to recall bias as we interviewed women who delivered up to 12 months prior to data collection. However, we primarily asked about what influences the location of delivery for "women in this community," which asks about social norm and is less subject to recall bias than personal experiences and decision-making.[48] Second, it is likely that the presence of this study resulted in increased awareness of MWHs among control populations, as shown by the main quantitative study findings published separately (Scott NA, "If we build it, will they come? Results of a quasi-experimental trial assessing the impact of maternity waiting homes on facility-based childbirth and maternity care in Zambia", Manuscript submitted for publication). We conducted multiple rounds of data collection for both the impact (baseline and endline) and implementation studies where community members and health system staff were asked about MWHs. Third, we did not disaggregate by MWH users and non-users or by location of MWH stay or delivery location. All these personal experiences likely influenced women's responses. We sought to understand how community-level perceptions changed over time, not just individual-level decision-making. A different analysis could be conducted to understand responses from women based on their MWH use or delivery location. Lastly, our intervention was implemented in a specific policy environment, and in SMGL-supported districts, limiting generalizability beyond the study districts.

For peer review only - http://bmjopen.bmj.com/site/about/guidelines.xhtml

CONCLUSION

MWHs can help to address some of the perceived barriers to facility-based delivery; however other obstacles persist. While MWH may be useful to address the distance barriers, no single intervention is likely to address all the barriers experienced by remote, rural, and poor populations. The quantitative and qualitative results from this study suggest MWHs could be considered in a broader systems approach to improving access in remote areas.

Contributions

Co-principal investigators NAS and JRL acquired funding and conceived the research question and designed the trial. NAS, JLK, RMF, TV, and TN oversaw investigation, research methodology, and data acquisition. JLK, RMF, TN, MB, and VRS were responsible for data curation. RMF conducted the data analysis. TN, GB, and GM participated in study coordination provincially and nationally. MB, VRS, and GM implemented the interventions and oversaw project administration. RMF, JLK, KJK, TV, and NAS wrote the original draft of this manuscript. All authors reviewed and edited the manuscript and approved the final version.

Funding statement

This program was developed and implemented in collaboration with Merck for Mothers, Merck's 10-year, $500 million initiative to help create a world where no woman dies giving life. Merck for Mothers is known as MSD for Mothers outside the United States and Canada (MRK 1846-06500.COL). The development of this article was also supported in part by the Bill & Melinda Gates Foundation (OPP1130334) https://www.gatesfoundation.org/How-We-Work/Quick-Links/Grants-Database/Grants/2015/06/OPP1130334 and The ELMA Foundation (ELMA-15-F0010) http://www.elmaphilanthropies.org/the-elma-foundation/. The funders had no role in study design,

For peer review only - http://bmjopen.bmj.com/site/about/guidelines.xhtml

data collection and analysis, decision to publish, or preparation of the manuscript. The content is solely the responsibility of the authors and does not necessarily reflect positions or policies of Merck, the Bill & Melinda Gates Foundation, or The ELMA Foundation. Funding for Online Open publication supported by Chronos Support through the Bill & Melinda Gates Foundation.

Declaration of Interest

All authors report their institutions received grants directly or indirectly from the Bill & Melinda Gates Foundation, MSD for Mothers, and The ELMA Foundation to conduct this study.

Acknowledgments

We appreciate the efforts, commitment, and contributions of the project staff, administrative support staff, the data collection team, and interviewers. From the Boston University/Right to Care team, we are thankful for the contributions of Denson Chongwe, David Kalaba, Deophine Bwalya, Parker Chastain, Allison Juntunen, Kathleen McGlasson, Elizabeth Ragan, Carey Howard, and Jason Park. From the Africare/University of Michigan team, we appreciate the efforts of Anne Naggyi, Isaac Sakala, Jessy Mtenje, Elizabeth Simwawa, Tenford Phiri, Lupiya Chilambwe, Jameson Kaunda, Nchimunya Chiboola and Nancy Lockhart. We are thankful to the team of translators and transcribers, and the two qualitative data coders Ingrid Olson and Jordan Musante for their hard work. We acknowledge the ongoing support of the Zambian Ministry of Health, the Provincial Health Offices, the District Health Offices; we thank Mary Nambao for her support and advice. We thank the Chiefs who welcomed us into their communities. This study could not have happened without the tireless efforts of the health facility staff and community-based volunteers who were champions of the intervention, advisors to project staff, and community guides to data collection teams. Finally, we thank the women, their infants, and their families for welcoming data collection teams into their homes and participating in this study. This

For peer review only - http://bmjopen.bmj.com/site/about/guidelines.xhtml

research was conducted with funding from MSD for Mothers, the Bill & Melinda Gates Foundation, and The ELMA Foundation. The findings and conclusions are those of the authors and do not necessarily reflect the policies or positions of the funders.

Data Sharing Statement

De-identified data for this analysis will be made available through OpenBU. The OpenBU repository has data access policies and procedures consistent with NIH data sharing policies.

For peer review only

References

1 World Health Organization. WHO recommendations on health promotion interventions for maternal and newborn health. Geneva, Switzerland: 2015. doi:10.1017/CBO9781107415324.004

2 Wong KLM, Benova L, Campbell OMR. A look back on how far to walk: Systematic review and meta-analysis of physical access to skilled care for childbirth in Sub-Saharan Africa. *PLoS One* 2017;**12**:e0184432. doi:10.1371/journal.pone.0184432

3 Sacks E, Vail D, Austin-Evelyn K, *et al.* Factors influencing modes of transport and travel time for obstetric care: a mixed methods study in Zambia and Uganda. *Health Policy Plan* 2016;**31**:293–301. doi:10.1093/heapol/czv057

4 Kyei-Nimakoh M, Carolan-Olah M, McCann T V. Access barriers to obstetric care at health facilities in sub-Saharan Africa-a systematic review. *Syst Rev* 2017;**6**:1–16. doi:10.1186/s13643-017-0503-x

5 Sialubanje C, Massar K, Hamer DH, *et al.* Understanding the psychosocial and environmental factors and barriers affecting utilization of maternal healthcare services in Kalomo, Zambia: A qualitative study. *Health Educ Res* 2014;**29**:521–32. doi:10.1093/her/cyu011

6 Stekelenburg J, Kyanamina S, Mukelabai M, *et al.* Waiting too long: Low use of maternal health services in Kalabo, Zambia. *Trop Med Int Heal* 2004;**9**:390–8. doi:10.1111/j.1365-3156.2004.01202.x

7 Fogliati P, Straneo M, Mangi S, *et al.* A new use for an old tool: maternity waiting homes to improve equity in rural childbirth care. Results from a cross-sectional hospital and community survey in Tanzania. *Health Policy Plan* 2017;**32**:1354–60. doi:10.1093/heapol/czx100

8 Republic of Zambia Ministry of Health. National Health Strategic Plan 2011 - 2015. Lusaka, Zambia: 2010.

9 Republic of Zambia Ministry of Health. National Health Strategic Plan 2006-2010. Lusaka, Zambia: 2005.

10 Masiye F, Chitah BM, McIntyre D. From targeted exemptions to user fee abolition in health care: Experience from rural Zambia. *Soc Sci Med* 2010;**71**:743–50. doi:10.1016/j.socscimed.2010.04.029

11 Ministry of Health (MOH) Zambia, Ministry of Community Development Mother and Child Health (MCDMCH) Zambia. Roadmap for Accelerating Reduction of Maternal , Newborn and Child Mortality, 2013-2016. 2013.

12 Morrissey Conlon C, Serbanescu F, Marum L, *et al.* Saving Mothers , Giving Life: It takes a system to save a mother. *Glob Heal Sci Pract* 2019;**7**:S6–26. doi:https://doi.org/10.9745/GHSP-D-18-00427

13 Serbanescu F, Clark TA, Goodwin MM, *et al.* Impact of the SMGL approach on decreasing maternal and perinatal deaths in Uganda and Zambia. *Glob Heal Sci Pract* 2019;**7**:S29–47. doi:https://doi.org/10.9745/GHSP-D-18-00428

14 Henry EG, Semrau K, Hamer DH, *et al.* The influence of quality maternity waiting homes on utilization of facilities for delivery in rural Zambia. *Reprod Health* 2017;**14**:68. doi:10.1186/s12978-017-0328-z

15 Lori JR, Wadsworth AC, Munro ML, *et al.* Promoting access: The use of maternity waiting homes to achieve safe motherhood. *Midwifery* 2013;**29**:1095–102. doi:10.1016/j.midw.2013.07.020

16 Stekelenburg J, Van Lonkhuijzen L, Spaans W, *et al.* Maternity waiting homes in rural districts in Africa: A cornerstone of safe motherhood? *Curr Womens Health Rev* 2006;**2**:235–8. doi:10.2174/157340406778699914

17 Penn-Kekana L, Pereira S, Hussein J, *et al.* Understanding the implementation of maternity waiting homes in low- and middle-income countries: a qualitative thematic synthesis. *BMC Pregnancy Childbirth* 2017;**17**:269. doi:10.1186/s12884-017-1444-z

18 Gabrysch S, Cousens S, Cox J, *et al.* The influence of distance and level of care on delivery place in rural Zambia: a study of linked national data in a geographic information system. *PLoS Med* 2011;**8**. doi:10.1371/journal.pmed.1000394

19 Scott NA, Henry EG, Kaiser JL, *et al.* Factors affecting home delivery among women living in remote areas of rural Zambia : a cross-sectional, mixed-methods analysis. *Int J Womens Health* 2018;**10**:589–601.

20 Lori JR, Munro ML, Rominski S, *et al.* Maternity waiting homes and traditional midwives in rural Liberia. *Int J Gynecol Obstet* 2013;**123**:114–8. doi:10.1016/j.ijgo.2013.05.024

21 Perosky JE, Munro-Kramer ML, Lockhart N, *et al.* Maternity waiting homes as an intervention to increase facility delivery in rural Zambia. *Int J Gynecol Obstet* 2019;**146**:266–7. doi:10.1002/ijgo.12864

22 Scott NA, Vian T, Kaiser JL, *et al.* Listening to the community: Using formative research to strengthen maternity waiting homes in Zambia. *PLoS One* 2018;**13**:e0194535. doi:https://doi.org/10.1371/journal.pone.0194535

23 Lori JR, Munro-Kramer ML, Mdluli EA, *et al.* Developing a community driven sustainable model of maternity waiting homes for rural Zambia. *Midwifery* 2016;**41**:89–95. doi:10.1016/j.midw.2016.08.005

For peer review only - http://bmjopen.bmj.com/site/about/guidelines.xhtml

24 Lori J, Munro-Kramer M, Liu H, *et al.* Increasing facility delivery through maternity waiting homes for women living far from a health facility in rural Zambia: a quasi-experimental study. *Br J Obstet Gynaecol* Published Online First: 2021. doi:10.1111/1471

25 Scott NA, Kaiser JL, Vian T, *et al.* Impact of maternity waiting homes on facility delivery among remote households in Zambia: protocol for a quasiexperimental, mixed-methods study. *BMJ Open* 2018;**8**. doi:10.1136/bmjopen-2018-022224

26 Central Statistical Office (CSO) Zambia. Zambia 2010 Census of Population and Housing National Analytical Report. 2012.

27 Central Statistical Office Zambia. 2015 Living Conditions Monitoring Survey Report. 2016. http://www.zamstats.gov.zm/report/Lcms/2006-2010 LCMS Report Final Output.pdf

28 Greeson D, Sacks E, Masvawure TB, *et al.* Local adaptations to a global health initiative: penalties for home births in Zambia. *Health Policy Plan* 2016;:czw060. doi:10.1093/heapol/czw060

29 Dobility INC. SurveyCTO [Technology for digical data collection]. 2017.

30 Gale NK, Heath G, Cameron E, *et al.* Using the framework method for the analysis of qualitative data in multi-disciplinary health research. *BMC Med Res Methodol* 2013;**13**:117. doi:10.1186/1471-2288-13-117

31 Ritchie J, Lewis J. *Qualitative research practice: a guide for social science students and researchers*. London: 2003. doi:March 10, 2016

32 O'Brien BC, Harris IB, Beckman TJ, *et al.* Standards for reporting qualitative research: a synthesis of recommendations. *Acad Med* 2014;**89**:1245–51. doi:10.1097/ACM.0000000000000388

33 Becker MH. The Health Belief Model and Personal Health Behavior. *Health Educ Monogr* 1974;**2**:324–508.http://dx.doi.org/10.1177/109019817400200407

34 Janz NK, Becker MH. The Health Belief Model: a decade later. *Health Educ Q* 1984;**11**:1–47. doi:10.1177/109019818401100101

35 Vian T, Fong RM, Kaiser JL, *et al.* Using Open Public Meetings and Elections to Promote Inward Transparency and Accountability: Lessons From Zambia. *Int J Heal Policy Manag* 2020;:1–13. doi:10.34172/ijhpm.2020.84

36 Kaiser JL, Fong RM, Ngoma T, *et al.* The effects of maternity waiting homes on the health workforce and maternal health service delivery in rural Zambia: A qualitative analysis. *Hum Resour Health* 2019;**17**. doi:10.1186/s12960-019-0436-7

37 Fontanet CP, Fong RM, Kaiser JL, *et al.* A qualitative exploration of community ownership of a maternity waiting home model in rural zambia. *Glob Heal Sci Pract* 2020;**8**:344–57. doi:10.9745/GHSP-D-20-00136

38 Sakanga VI, Chastain P, McGlasson KL, *et al.* Building financial management capacity for community ownership of development initiatives in rural Zambia. *Int J Health Plann Manage* 2019;**34**. doi:10.1002/hpm.2810

39 Sialubanje C, Massar K, G van der Pijl MS, *et al.* Improving access to skilled facility-based delivery services: Women's beliefs on facilitators and barriers to the utilisation of maternity waiting homes in rural Zambia. *Reprod Health* 2015;**12**. doi:10.1186/s12978-015-0051-6

For peer review only - http://bmjopen.bmj.com/site/about/guidelines.xhtml

40 Republic of Zambia Ministry of Health. The 2012 List of Health Facilities in Zambia. 2013. http://www.moh.gov.zm/docs/facilities.pdf (accessed 31 May 2018).

41 World Health Organization. Global Reference List of 100 Core Health Indicators. Geneva, Switzerland: 2015. apps.who.int/iris/bitstream/10665/173589/1/WHO_HIS_HSI_2015.3_eng.pdf%0Ahttp://ci.nii.ac.jp/naid/40020358184/

42 Moyer CA, Mustafa A. Drivers and deterrents of facility delivery in sub-Saharan Africa: a systematic review. *Reprod Health* 2013;**10**:1–14. doi:10.1186/1742-4755-10-40

43 Bohren MA, Hunter EC, Munthe-Kaas HM, *et al.* Facilitators and barriers to facility-based delivery in low- and middle-income countries: a qualitative evidence synthesis. *Reprod Health* 2014;**11**:71. doi:10.1186/1742-4755-11-71

44 Chibuye PS, Bazant ES, Wallon M, *et al.* Experiences with and expectations of maternity waiting homes in Luapula Province, Zambia: a mixed–methods, cross-sectional study with women, community groups and stakeholders. *BMC Pregnancy Childbirth* 2018;**18**:42. doi:10.1186/s12884-017-1649-1

45 Kaiser JL, Mcglasson KL, Rockers PC, *et al.* Out-of-pocket expenditure for home and facility- based delivery among rural women in Zambia : a mixed-methods, cross-sectional study. *Int J Womens Health* 2019;**11**:411–30. doi:10.2147/IJWH.S214081

46 Chiu C, Scott NA, Kaiser JL, *et al.* Household saving during pregnancy and facility delivery in Zambia: a cross-sectional study. *Health Policy Plan* 2019;:1–8. doi:10.1093/heapol/czz005

47 Gaym A, Pearson L, Soe KWW. Maternity waiting homes in Ethiopia--three decades experience. *Ethiop Med J* 2012;**50**:209–19.

48 Miles MB, Huberman AM. *Qualitative Data Analysis: An Expanded Sourcebook, 2nd Edition*. 2nd ed. Thousand Oaks, California: : SAGE Publications, Inc 1994.

For peer review only - http://bmjopen.bmj.com/site/about/guidelines.xhtml

BMJ Open

Barriers and facilitators to facility-based delivery in rural Zambia: A qualitative study of women's perceptions after implementation of an improved Maternity Waiting Homes intervention

Journal:	*BMJ Open*
Manuscript ID	bmjopen-2021-058512.R1
Article Type:	Original research
Date Submitted by the Author:	11-Mar-2022
Complete List of Authors:	Fong, Rachel; Boston University School of Public Health, Global Health<br>Kaiser, Jeanette; Boston University School of Public Health, Global Health<br>Ngoma, Thandiwe; Right to Care Zambia, Research<br>Vian, Taryn; University of San Francisco - School of Nursing and Health Professions<br>Bwalya, Misheck; mothers2mothers<br>Sakanga, Viviane; Amref Health Africa, Programmes<br>Lori, Jody; University of Michigan School of Nursing, Office for Global Affairs & PAHO/WHO Collaborating Center<br>Kuhfeldt, Kayla; Boston University School of Public Health, Global Health<br>Musonda, Gertrude; Africare Zambia<br>Munro-Kramer, Michelle; University of Michigan School of Nursing, Health Behavior & Biological Sciences<br>Rockers, Peter; Boston University School of Public Health, Department of Global Health<br>Hamer, D.; Boston University School of Public Health, Department of Global Health<br>Ahmed Mdluli, Eden; Project Hope<br>Biemba, Godfrey; National Health Research Authority, Pediatric Centre of Excellence<br>Scott, Nancy; Boston University School of Public Health, Global Health
**Primary Subject Heading**:	Global health
Secondary Subject Heading:	Health services research, Public health, Qualitative research
Keywords:	QUALITATIVE RESEARCH, PUBLIC HEALTH, Health policy < HEALTH SERVICES ADMINISTRATION & MANAGEMENT

SCHOLARONE™
Manuscripts

I, the Submitting Author has the right to grant and does grant on behalf of all authors of the Work (as defined in the below author licence), an exclusive licence and/or a non-exclusive licence for contributions from authors who are: i) UK Crown employees; ii) where BMJ has agreed a CC-BY licence shall apply, and/or iii) in accordance with the terms applicable for US Federal Government officers or employees acting as part of their official duties; on a worldwide, perpetual, irrevocable, royalty-free basis to BMJ Publishing Group Ltd ("BMJ") its licensees and where the relevant Journal is co-owned by BMJ to the co-owners of the Journal, to publish the Work in this journal and any other BMJ products and to exploit all rights, as set out in our licence.

The Submitting Author accepts and understands that any supply made under these terms is made by BMJ to the Submitting Author unless you are acting as an employee on behalf of your employer or a postgraduate student of an affiliated institution which is paying any applicable article publishing charge ("APC") for Open Access articles. Where the Submitting Author wishes to make the Work available on an Open Access basis (and intends to pay the relevant APC), the terms of reuse of such Open Access shall be governed by a Creative Commons licence – details of these licences and which Creative Commons licence will apply to this Work are set out in our licence referred to above.

Other than as permitted in any relevant BMJ Author's Self Archiving Policies, I confirm this Work has not been accepted for publication elsewhere, is not being considered for publication elsewhere and does not duplicate material already published. I confirm all authors consent to publication of this Work and authorise the granting of this licence.

For peer review only

Barriers and facilitators to facility-based delivery in rural Zambia: A qualitative study of women's perceptions after implementation of an improved Maternity Waiting Homes intervention

Author Name	Highest Degree	Affiliation	Contact
Rachel M. Fong	MPH	Department of Global Health, Boston University School of Public Health, Boston, MA, USA	rmfong@bu.edu
Jeanette L. Kaiser	MPH	Department of Global Health, Boston University School of Public Health, Boston, MA, USA	jlkaiser@bu.edu
Thandiwe Ngoma	MPH	Department of Research, Right to Care Zambia, Lusaka, Zambia	thandiwe.ngoma@equiphealth.org
Taryn Vian	PhD	School of Nursing and Health Professions, University of San Francisco, San Francisco, CA, USA	tvian@usfca.edu
Misheck Bwalya	MSc	mothers2mothers, Lusaka, Zambia	bwalyamisheck@gmail.com
Viviane R Sakanga	MPH	Amref Health Africa in Zambia, Lusaka, Zambia	Vivianesakanga@gmail.com
Jody R. Lori	PhD	Office for Global Affairs & PAHO/WHO Collaborating Center, University of Michigan School of Nursing, Ann Arbor, MI USA	jrlori@umich.edu
Kayla J. Kuhfeldt	MPH	Department of Global Health, Boston University School of Public Health, Boston, MA, USA	kaylakuh@bu.edu
Gertrude Musonda	MPH	Africare Zambia, Lusaka, Zambia	gmusonda@africare.org
Michelle L. Munro-Kramer	PhD	Department of Health Behavior & Biological Sciences, University of Michigan School of Nursing, Ann Arbor, MI USA	mlmunro@umich.edu
Peter C. Rockers	ScD	Department of Global Health, Boston University School of Public Health, Boston, MA, USA	prockers@bu.edu
Davidson H. Hamer	MD	Department of Global Health, Boston University School of Public Health; Section of Infectious Diseases, Department of Medicine, Boston University School of Medicine, Boston, MA USA	dhamer@bu.edu
Eden Ahmed Mdluli	MPH	Project HOPE	edenamm@gmail.com

For peer review only - http://bmjopen.bmj.com/site/about/guidelines.xhtml

		7500 Old Georgetown Rd<br>Bethesda, MD 20814	
Godfrey Biemba	MSc.	National Health Research Authority, Pediatric Centre of Excellence, Lusaka, Zambia	gbiemba@gmail.com
Nancy A. Scott	DrPH	Department of Global Health, Boston University School of Public Health, Boston, MA, USA	nscott@bu.edu

Corresponding Author:

Rachel M Fong
801 Massachusetts Avenue
Crosstown Center, 3rd Floor
Boston, MA 02118 USA
Email: rmfong@bu.edu
Phone: 617-358-2184

For peer review only

For peer review only - http://bmjopen.bmj.com/site/about/guidelines.xhtml

ABSTRACT

Objectives: Women in sub-Saharan Africa face well-documented barriers to facility-based deliveries. An improved maternity waiting homes (MWH) model was implemented in rural Zambia to bring pregnant women closer to facilities for delivery. We qualitatively assessed whether MWHs changed perceived barriers to facility delivery among remote-living women.

Design: We administered in-depth interviews (IDIs) to a randomly-selected subsample of women in intervention (n=78) and control (n=80) groups who participated in the primary quasi-experimental evaluation of an improved MWH model. The IDIs explored perceptions and preferences of delivery location. We conducted content analysis to understand perceived barriers and facilitators to facility delivery.

Setting and participants: Participants lived in villages 10+ kilometers from the health facility and had delivered a baby in the previous 12 months.

Intervention: The improved MWH model was implemented at 20 rural health facilities.

Results: Over 96% of participants in the intervention arm and 90% in the control arm delivered their last baby at a health facility. Key barriers to facility delivery were distance and transportation, and costs associated with delivery. Facilitators included no user fees, penalties for home delivery, desire for safe delivery, and availability of MWHs. Most themes were similar between study arms. Both discussed the role MWHs have in improving access to facility-based delivery. Intervention arm participants expressed that the improved MWH model encourages use and helps overcome the distance barrier. Control arm participants either expressed a desire for an improved MWH model or did not consider it in their decision-making.

Conclusions: Even in areas with high facility-based delivery rates in rural Zambia, barriers to access persist. MWHs may be useful to address the distance challenge, but no single intervention is likely to address all barriers experienced by rural, low-resourced populations. MWHs should be considered in a broader systems approach to improving access in remote areas.

Trial Registration: ClinicalTrials.gov Identifier: NCT02620436

Key words: Mothers' shelter; Maternal health; Qualitative research; Community perceptions; Health behaviors

ARTICLE SUMMARY

Strengths and limitations of this study

1. The study was conducted after the implementation of a large-scale maternity waiting home intervention, which allows us to examine the differences in women's perceptions of barriers experienced between areas that received the Core Maternity Waiting Home Model and areas that did not receive the intervention.
2. We used a randomly selected, representative sample of the population, which is rare in qualitative research, and it assured the best opportunity to capture a diversity of perceptions.

For peer review only - http://bmjopen.bmj.com/site/about/guidelines.xhtml

3. Our sample of women living in rural Zambia is a hard-to-reach population that likely face the greatest barriers to facility delivery due to their low socio-economic status, poor road infrastructure, limited transportation options, and low densities of facilities and skilled birth attendants.
4. The findings are subject to recall bias, as we interviewed women who delivered up to 12 months prior to data collection, however, topics of discussion surrounded social norm, which is less subject to recall bias than personal experiences and decision-making.
5. We conducted multiple rounds of data collection throughout the duration of the study, which likely resulted in increased awareness of maternity waiting homes among the control groups.

For peer review only

For peer review only - http://bmjopen.bmj.com/site/about/guidelines.xhtml

INTRODUCTION

To reduce maternal and neonatal mortality, the World Health Organization (WHO) recommends that every birth occurs at an equipped health facility, attended by a skilled professional.[1] However, home births remain common in many areas, particularly in rural, low-resource settings. Individual and system level factors associated with births outside of equipped health facilities are well documented and include lack of access to facilities, long distances, lack of transportation, costs associated with delivery, and women's decision-making autonomy.[2–7]

The Government of the Republic of Zambia (GRZ) has sought to increase facility-based deliveries and reduce maternal mortality through an increasingly articulated policy landscape, monitoring of relevant indicators, and strategic partnerships to impact supply and demand of maternal health services. Within its National Health Strategic Plan (NHSP), which is published approximately every five years and sets out the country's health goals over that period, the GRZ has emphasized the importance of deliveries supervised by skilled health personnel. This has been included as a monitoring indicator since at least 2005, and subsequently as a main objective of the NHSP since at least 2010.[8,9] Additionally, in 2006, the GRZ eliminated user fees on primary health services, including maternal and child health services, first in rural areas then countrywide, to remove cost barriers and increase universal access to health services.[10]

The GRZ more clearly articulated its commitment to reducing maternal mortality and increasing maternal health more generally through its "Roadmap for accelerating the reduction of maternal, newborn, and child mortality," which mapped all stakeholder groups and organizations within the country and set out a multipronged, ambitious plan for achieving its goals.[11] Through the Saving Mothers, Giving Life (SMGL) initiative, formed in 2011, which combined GRZ efforts with multiple external funders and implementers, many of the plans set out in the roadmap were implemented. Between 2012 and 2016, SMGL significantly increased facility-based delivery rates from 63% to 90% and

For peer review only - http://bmjopen.bmj.com/site/about/guidelines.xhtml

reduced maternal mortality by approximately 40% in multiple districts of Zambia through intensive investments in increasing access to and improving quality of health services, care coordination, and community outreach programs.[12,13] These same results were not experienced in non-SMGL-supported districts.[12]

One strategy to increase facility-based deliveries is maternity waiting homes (MWHs), which in theory allow a pregnant woman to reside near a health facility in the weeks prior to her estimated delivery date.[7,14–16] With lower densities of equipped health facilities in rural areas, and poor road infrastructure, women residing in rural locations typically endure lengthy travel to deliver at a well-equipped health facility and often experience challenges accessing transportation.[17–19] In rural settings, the presence of quality MWHs has been associated with increased rates of facility-based delivery.[14,20–22]

Through formative research conducted in Zambia, the Maternity Homes Alliance (MHA), a partnership between the GRZ, two local non-governmental organizations (Right to Care Zambia and Africare), and two academic institutions (Boston University and the University of Michigan), designed a Core MWH Model targeting pregnant women living most remotely.[23,24] The MHA hypothesized that quality MWHs could remove distance barriers to facility delivery by allowing women to travel to facilities earlier than when they are in labor or when it becomes urgent. The MHA constructed 20 MWHs following the Core MWH Model (referred to hereafter as the Core MWH Model) and evaluated the impact on facility delivery rates among women living more than 10 kilometers from their designated rural health facility.[25] The Core MWH Model was associated with increased rates of facility-based delivery and MWH utilization among remote-living women.[22,26] The MHA also evaluated the implementation effectiveness of the Core MWH Model to assess implementation fidelity and factors that affect adoption, uptake, and sustainability of the intervention.[27] A mixed-methods sub-study looking at the quality and utilization of MWHs at referral facilities found that the Core MWH Model had

For peer review only - http://bmjopen.bmj.com/site/about/guidelines.xhtml

a significantly higher quality score than comparison site MWHs, and there was an increase in MWH utilization at both intervention and comparison sites, though there was a higher increase at one of the two intervention sites, after the implementation of the intervention.[28] As part of the larger impact evaluation, this study aimed to understand how the Core MWH Model influenced maternal perceptions of barriers and facilitators to facility-based delivery by conducting a qualitative assessment among women living in villages 10+ kilometers from the health facility in catchment areas that received the Core MWH Model compared to those living in control catchment areas without the improved MWHs.

METHODS

Study setting

This study was conducted in seven rural districts in Zambia which received the SMGL intervention: Nyimba and Lundazi in Eastern Province; Choma, Kalomo and Pemba in Southern Province; and Mansa and Chembe in Luapula Province.[25] These districts are generally rural, with low population densities, and their main industries are agriculture.[29] The majority of residents, especially away from the few urban centers, are subsistence farmers who may make some additional income selling crops or doing piecework (short-term, casual work).[30]

The mixed methods baseline evaluation for this study found high facility delivery rates of 81.4% among women living more than 10 km from their rural health facility, with 15.3% of women still delivering at home and 3.2% delivering on the way to a health facility.[19] Qualitatively, these remote-living women discussed transport availability, distance, and cost of transport and supplies as continued barriers to facility-based deliveries. They also noted informal penalty fees being levied by traditional leaders against women who delivered at home, which had been previously reported in the literature.[19,31]

For peer review only - http://bmjopen.bmj.com/site/about/guidelines.xhtml

Intervention description

Described fully elsewhere, the MHA constructed 20 MWHs that met each of the three main pillars of the Core MWH Model:

(1) Infrastructure, equipment and supplies to ensure a safe, comfortable, and functional structure. The Core MWH Model is a multi-room structure with concrete floors and walls, a secure roof, and lockable doors and windows. The structure includes residential space for pregnant women, a separate pace for postnatal women and newborns, latrines, a bathing area, a cooking space, and a veranda for socializing and education sessions. Amenities include lighting, cooking supplies, beds, bedding, mosquito nets, and storage space.

(2) Policies, management, and finances to ensure local oversight and sustainability of the homes. Each MWH has a management unit and a governance committee which includes members of the community, health facility staff, and local government. These oversight organizations oversee daily operations, and maintain financial well-being and sustainability of the MWH.

(3) Linkages and services to ensure integration with the formal health system. All clinical care was provided at the health facility, but health facility staff jointly oversaw the MWHs as part of its governing committee and checked in daily on waiting women. Education courses on maternal and child health topics were conducted weekly at each intervention site.[25]

The implementation of the Core MWH Model in all 20 sites was similar, with minor differences in how the partners and individual sites operationalized the policies, management, and finances pillar. For example, income-generating activities--such as maize grinding, goat raising, or a store--were implemented at intervention sites to help finance and sustain the MWHs; each site implemented its own income-generating activity.

Twenty rural health facilities implementing the standard of care for waiting women were selected as matched control sites.[25] The standard of care varied widely with some having no formal

For peer review only - http://bmjopen.bmj.com/site/about/guidelines.xhtml

space for waiting women and others having a small, community-constructed MWH in generally poor condition and minimal amenities, if any.

Study design

Data for this analysis were collected from September to October 2018 as part of the endline data collection of the quasi-experimental, cluster-controlled impact evaluation.[25,32,33] A household survey was administered to a randomly selected cross-section of 2,330 participants, 1,217 in the intervention, 1,113 in the control sites who lived 10 or more kilometers from their designated rural health facility. A randomly selected subsample of participants (7%) also completed an in-depth interview (IDI) following the survey. The semi-structured IDI guide was based off of the household survey questions (Supplementary File 1). It had a series of open-ended questions with prompts to explore themes on personal and community perceptions of factors that influence delivery location, quality of MWHs, factors that influence utilization of MWHs, planning for delivery, and costs related to delivery. The questions included probes for various themes that arose during baseline data collection and known facilitators and barriers from existing literature, including culture, cost, distance, transportation, safety, comfort, and local laws, among others.[19] The intervention MWHs were launched shortly after baseline data collection (March to May 2016) of the impact evaluation and operated for a minimum of 13 months before endline data collection.

Participants

A random sample of participants were asked to participate in the IDI following their household survey. Per the primary study eligibility criteria, all participants were at least 15 years of age or older and delivered a baby in the past 12 months, irrespective of location of delivery. To mitigate selection bias, we first randomly selected four villages per catchment area, then randomly selected one household in each village to participate in the IDI following the main survey. This helped to select a representative sample of women in the sample frame.[25]

For peer review only - http://bmjopen.bmj.com/site/about/guidelines.xhtml

Data collection

Data collectors fluent in English and the four relevant local languages were trained in human subjects' protection, electronic data collection using Android tablets, and qualitative research methods. Immediately following the quantitative household survey, selected participants were offered a short break before commencing the IDI, which took approximately 30 minutes to complete. All interviews were audio recorded with consent from the participant. Participant demographics, collected during the household survey, were linked to IDI participants through their unique study ID. Demographics were captured electronically on encrypted tablets using the SurveyCTO Collect software (Dobility, Inc, Cambridge, MA).[34]

Data management and analysis

All IDI recordings were translated and transcribed verbatim into Microsoft® Word. The transcripts were coded line by line in NVivo v12 (QSR International, Doncaster, Australia) by two research staff familiar with the context and study. [33,35,36] The main codes were created *a priori* using the established themes from the interview guide. Additional sub-codes were added during coding as themes emerged. The first few transcripts were coded by both research staff and a coding comparison was run in NVivo v12 (QSR International, Doncaster, Australia) using a kappa coefficient to mitigate researcher bias. Codes with poor agreement (kappa value <0.40) were discussed and agreed upon. Codes were reviewed throughout the coding process and merged if there was agreement that two or more codes contained similar content. Discrepancies were deliberated and resolved. Matrix queries in NVivo v12 (QSR International, Doncaster, Australia) were used during the analysis to organize and understand themes in the coded data to compare perceived facilitators and barriers to facility delivery between remote, rural women living in catchment areas with the Core MWH Model and those in areas without the Core MWH Model. The Health Belief Model explains health behaviors as the presence or absence of multiple constructs, including perceived benefits and barriers to a behavior.[37] Themes with

For peer review only - http://bmjopen.bmj.com/site/about/guidelines.xhtml

more than 10 participants (25% of the sample) were summarized and included in the results. Findings were triangulated within the context of the bigger impact evaluation. Quotations which included costs in Zambian Kwacha (K) were converted to US Dollars (US$) using an approximate average exchange rate over the 12 months preceding data collection (K10 to US$1). Findings were reported using the Standards for Reporting Qualitative Research Checklist.[38]

Demographic data were cleaned and analyzed in SAS v9.4 (SAS Institute Inc., Cary, NC, USA).[32] We present demographics for the recently delivered women and their households, and the women's primary outcomes of delivery at a health facility. We report the median and interquartile range (IQR) for non-normal data, means and standard deviations (SD) or proportions where appropriate.

Patient and Public Involvement

Key community members including women, men, elders and traditional leadership were consulted in the conceptualization and design of the Core MWH Model.[23,24] The final design also included input from stakeholders at all levels throughout the Ministry of Health. Through a process evaluation during implementation, end-users and the public were regularly consulted to understand the acceptability and sustainability of the intervention.[39–42]

Ethical considerations

Ethical approvals for this study were obtained from the Boston University Medical Campus Institutional Review Board (IRB; Protocol number H-34526) and the ERES Converge IRB in Zambia (Protocol number 2015-Dec-012). The Zambian Ministry of Health and National Health Research Authority granted approval. Additionally, the Provincial Health Officers, and the District Health Officer in each study district supported the study. Written informed consent was obtained from all participants prior to conducting the household survey and IDI. Assent and guardian consent were obtained, when necessary, from participants 15 years old and younger. Trial Registration Number: NCT02620436

For peer review only - http://bmjopen.bmj.com/site/about/guidelines.xhtml

RESULTS

Participant Characteristics

Demographic characteristics for the 158 IDI participants were similar between the intervention and control groups (Table 1). Participants in both arms lived a similar distance from their assigned health facility (12-13 km) and had about 6 years of education. More intervention participants (65.4%) reported having used a MWH than in the control arm (42.5%). More participants in the intervention arm delivered at a health facility (96.1%) compared to the control arm (90.0%).

Table 1. Qualitative participant characteristics for the endline observation of the MWH impact study, by study arm

	Intervention n = 78	Control n= 80
**Participant characteristics**		
**Woman's age in years**, median (IQR)	26 (21, 32)	29.5 (23, 35)
**Years of education**, mean (SD)	6.1 (3.2)	6.0 (3.4)
**Married/Cohabiting**, n (%)	68 (87.2)	71 (88.7)
**Gravida**, mean (SD)	4.0 (2.4)	4.5 (2.3)
**Parity,** mean (SD)	3.5 (2.4)	4.2 (2.3)
**Primigravida**, n (%)	16 (20.5)	10 (12.5)
**Four or more ANC visits**, n (%)	60 (75.0)	59 (75.6)
**Delivered at health facility or hospital**, n (%)	75 (96.1)	72 (90.0)
**Age of most recently delivered baby (months)**, mean (SD)	7.0 (3.7)	7.1 (3.6)
**District**, n (%)		
**Choma/Pemba**	15 (19.2)	11 (13.7)
**Kalomo**	16 (20.5)	21 (26.2)
**Nyimba**	8 (10.3)	8 (10.0)
**Lundazi**	20 (25.6)	20 (25.0)
**Mansa/Chembe**	19 (24.4)	20 (25.0)
**Household Characteristics**		

For peer review only - http://bmjopen.bmj.com/site/about/guidelines.xhtml

**Household size**, median (IQR)	6 (5, 8)	7 (5, 8)
**Dependency Ratio** [a], mean (SD)	1.5 (1, 2)	1.5 (1, 2)
**Travel distance to health facility (km)**, median (IQR)	12.1 (11, 15)	13.2 (11, 16)
**No electricity**, n (%)	77 (98.7)	79 (99.0)
**Unimproved sanitation** [b], n (%)	66 (84.6)	59 (73.7)

Abbreviations: IQR = interquartile range; SD = standard deviation; ANC = antenatal care; MWH = maternity waiting home

[a] Dependency Ratio = (children <16 years old + adults >65 years old)/adults > 16 years old.

[b] Unimproved sanitation: pit latrine without slab/open pit, bucket toilet, hanging toilet/latrine, no facility/bush/field.

Factors influencing delivery location

Two major barriers influencing facility-based delivery emerged: 1) distance and transportation, and 2) costs associated with delivery, while four key facilitators emerged: 1) no user fees, 2) penalties for home delivery, 3) desire for safe delivery, and 4) availability of MWHs (Table 2).

Table 2. Key barriers and facilitators to facility-based delivery reported by qualitative participants during the endline observation of the MWH impact study, by study arm

Key Themes	Intervention	Control
**Barriers influencing facility delivery**	1. **Distance & transportation**: health facility is far, have to prepare transport and money, buy fuel in advance or walk a long way; bad roads<br>2. **Costs associated with delivery**: need to prepare money for transportation (costs more if distance is farther) and buy required delivery supplies<br>a. **Costs associated with MWH stay:** need to bring food to cook during stay<br>b. **Stigma related to costs:** worried, scared, or embarrassed about not having the delivery supplies required and/or baby clothes to bring to the health facility	1. **Distance & transportation**: health facility is far and transportation is challenging, have to plan to walk early and wait at the facility or MWH if there is one or need to prepare transport and money<br>2. **Costs associated with delivery**: need to prepare money for transportation and buy required delivery supplies<br>a. **Stigma related to costs:** scared or embarrassed about not having delivery requirements and/or baby clothes to bring to the health facility
**Facilitators influencing facility delivery**	1. **Costs associated with delivery**: no cost to deliver at a facility; no cost to stay at MWH<br>2. **Penalties for home delivery**: penalty fee for home delivery – pay the chief and health facility; penalty made women	1. **Costs associated with delivery**: no cost to deliver at a facility<br>2. **Penalties for home delivery**: penalty fees for home delivery – pay the chief and health facility; penalty made women fear home delivery

For peer review only - http://bmjopen.bmj.com/site/about/guidelines.xhtml

	fear home delivery though some believed it is a hoax<br>**3. Safe delivery**: concerned about complications at home and afraid of dying; safer to deliver at facility, where staff can help with complications<br>**4. MWH availability**: good to move to MWH before delivery to avoid transportation difficulties because health facility is far<br>a. Advised to stay by health facility staff at 8 months if woman lived far away<br>b. MWH is free and has amenities such as beds, blankets, and cooking supplies	**3. Safe delivery**: concerned about delivery complications at home, and afraid of dying from complications; safer to deliver at facility, where staff can help with complications<br>**4. MWH availability**: women chose to go to health facilities with MWH no matter how far; good for those who live far away from the facility – can rest before delivery; no MWH but want one in their area; MWH availability does not matter, women will go where they can deliver safely

Distance and transportation

Participants in both study arms regarded distance and transportation as key barriers when deciding whether or not to deliver at a health facility. Participants generally acknowledged the health facility is far, so they have to prepare transportation (e.g. booking a car or taxi, borrowing a bicycle or ox cart) and put aside money in advance (Table 3, quotes a-d). No facilitators relating to distance and transportation arose in either the intervention or control groups.

For peer review only

Table 3. Quotations illustrating key themes as barriers or facilitators to facility-based delivery as reported by qualitative participants during the endline observation of the MWH impact study, by study arm

Key Themes	Theme as Barrier vs. Facilitator to Facility Delivery	Intervention	Control
**Distance and transportation**	**Barrier**	a) "Yes, that's the reason women go to the MWH early, transport is a major challenge." – Woman, Pemba District<br><br>b) "[The health facility] is quite far. We walked there. I started off around 3pm and reached there at 7pm." – Woman, Lundazi District	c) "The distance from here to the clinic is quite long and transport here is a big challenge, because you need to have money to pay." – Woman, Pemba District<br><br>d) "The distance [to the health facility] is very big and transport is a problem..." – Woman, Lundazi District
	**Facilitator**	No themes emerged	No themes emerged
**Costs associated with delivery**	**Barrier**	e) "I thought about where I was going to find money to get what is required. They tell you exactly what you need to buy when going for antenatal checkups so that you can prepare." – Woman, Lundazi District	f) "The basic costs that we face are buying baby clothes, delivery kit, transport, and buying food when you stay at the clinic as you wait to deliver." - Woman, Lundazi District<br><br>g) "This is why people deliver at home, because most have no money to prepare for delivery at facilities and when you get there without these [required items], nurses shout at us a lot." – Woman, Nyimba District
	**Facilitator**	h) "[The health facility] does not charge. They only ask us to prepare what to use during delivery and for our children too." – Woman, Chembe District	i) "That's why we prefer delivering from a health facility where it is free than in the village where you have to pay the village headman and other people." – Woman, Chembe District
**Penalties for home delivery**	**Barrier**	No themes emerged	No themes emerged
	**Facilitator**	j) "If you deliver at home, like me, when you go to the health facility to have the baby checked you will need to pay K200 (~US$20) before your baby is checked. The nurses are	k) **"**We have our traditional headmen who have partnered with the health facilities. If you deliver at home, you will pay a goat to the chief and then when you go to the health facility, they will make you pay K100 (~US$10) to get the

For peer review only - http://bmjopen.bmj.com/site/about/guidelines.xhtml

		the ones who give these penalties." – Woman, Kalomo District	growth monitoring card [for the baby]. Those are the local rules that we do follow." – Woman, Lundazi District
**Safe delivery**	**Barrier**	No themes emerged	No themes emerged
	**Facilitator**	l) "Different things [like complications, can] happen. For me, I went to the hospital and thought the baby will die. The doctor put me on a drip so that I could get better. They acted fast and I was written a letter and transferred to a bigger hospital." - Woman, Lundazi District	m) "They [health facility staff] will help you when a baby is in breach position. They will find ways of delivering a normal baby. But if you deliver from home and it [a breach delivery] happens, by the time you go to look for transport, you would have been dead." – Woman, Mansa District
**MWH availability**	**Barrier**	No themes emerged	No themes emerged
	**Facilitator**	n) "We mostly use ox-carts, but it is always better to go early and stay at the MWH until the day you deliver. I personally decided early that I needed to go to the MWH in case of difficulties with transport arrangements." – Woman, Kalomo District<br><br>o) "I considered the MWH. If I go there without pots I will be helped because they have pots there so I can cook before I deliver. Bed and blankets were also given." – Woman, Choma District	p) "Usually that [MWH] is the first thing we look at. Due to long distances to the health facility in this area, women choose to go deliver at the clinics where there is a MWH, no matter the distance. Second, women know that if you are staying at the MWH, the health facility staff will be checking you regularly to see if you and the baby are ok and that you can deliver without any problems." – Woman, Lundazi District

For peer review only

For peer review only - http://bmjopen.bmj.com/site/about/guidelines.xhtml

Costs associated with delivery

Delivery-associated costs were a frequently cited barrier. Nuances to these costs, such as costs associated with having to bring food for their MWH stay before delivery and stigma related to not having enough money to purchase delivery supplies were also captured. Many participants reported that procuring delivery supplies posed an obstacle to a facility-based delivery (Table 3, quotes e and f). A few participants mentioned that one reason they still deliver at home is due to fear of getting embarrassed or shouted at by the health facility staff if they are unable to buy all the delivery requirements including bleach, a bucket, gloves, chitenge (traditional wrapping cloth), baby blanket, baby clothes, and nappies (Table 3, quote g). Similarly, a woman in the intervention group and a woman in the control group discussed how health facility staff ignore or give less attention to patients who had not procured the required supplies. However, participants acknowledged that the health facility does not charge for the delivery itself (Table 3, quote h) and there is no cost to stay at the MWH, which were frequently cited as a facilitator. Participants in both study arms perceived home delivery to cost more due to penalty fees, discussed further below (Table 3, quote i).

Penalties for home delivery

Penalties for home delivery arose as a factor that affects decision-making for delivery location. Many participants discussed fearing home delivery, because they would have to pay either or both the health facility and to the village chief (Table 3, quotes j and k). This factor emerged in both study arms and at all sites, though it was more salient for women in the control arm. Women reported payments to the chief in the form of a goat or chicken, and making monetary payments to the health facility staff (reported amounts between 5 – 25 U.S. Dollars, depending on the study site). Women believed the "law" came from either the government, the chief, or the health facility, and is enforced by the chief, health facility staff, and/or community health workers. When asked why the penalty exists, participants discussed preventing transmission of diseases such as HIV to the baby during delivery and preventing

For peer review only - http://bmjopen.bmj.com/site/about/guidelines.xhtml

maternal mortality. A few participants also reported having to pay for their child's growth monitoring card at the health facility when they went for postnatal care after delivering at home and considered this a "penalty" for their home delivery imposed by health facility staff. Attempts to avoid these penalties influenced women to delivery at health facilities.

Conversely, a few participants in the intervention arm believed the penalty fees for home delivery to be "a hoax" (a story that nurses pass around), because they had never seen it enforced in their communities. This belief appeared to diminish the effect of the penalties on increasing facility deliveries.

Safe delivery:

No barriers related to safe delivery arose in either the intervention or control groups. Despite the perceived barriers of distance, transportation, and cost, participants in both study arms regarded the health and well-being of the mother and baby as one of the top priorities for deciding where to deliver. Participants in both study arms emphasized the importance of delivering at a health facility as a facilitator, where trained health staff can help if any complications arose and expressed fear of dying from giving birth in their village (Table 3, quotes l and m). Many women gave examples of complications such as the positioning of the baby (breach) or losing blood, and they anticipated that the health facility had or would take care of them, give them blood or put them on "a drip." Some participants added that if the health facility staff were not able to handle the complications, they would be able to refer women to a higher-level facility.

MWH Availability:

No barriers related to MWH availability arose in either the intervention or control groups. Participants in both study arms acknowledged that people in their communities consider the availability of an MWH when deciding where to deliver, especially if they live very remotely (Table 3, quote n). This was more frequently discussed as a facilitator by participants in the intervention arm, where women

For peer review only - http://bmjopen.bmj.com/site/about/guidelines.xhtml

also noted the importance of the amenities provided at the MWH, such as beds, blankets, and cooking utensils (Table 3, quote o). Although some participants in the control arm noted the availability of a MWH in their area, many wished for one of higher quality. Some participants even expressed their willingness to travel farther to a health facility with a MWH (Table 3, quote p). However, some participants in the control arm reported they do not consider a MWH when deciding where to deliver; rather they are willing to travel to wherever they can deliver safely regardless of whether a MWH is present.

DISCUSSION

This qualitative study explored differences in the factors remote-living women perceive as influencing the location of delivery in communities randomly assigned to receive the Core MWH model intervention and in communities randomly assigned to standard of care. We found that the availability and characteristics of the Core MWH Model did seem important to pregnant women: the MWH was more frequently mentioned as a facilitator by participants in the intervention arm, and women emphasized the amenities provided at the Core MWH Model, such as beds, blankets, and cooking supplies.

At the same time, we found that some facilitators influencing delivery location were similar across both study arms. These included the perception of having access to a safer delivery, no user fees, and avoiding penalties for home delivery. These findings are supported by other literature emerging from the same areas in Zambia. [19,23,24,43]

Despite implementation of the Core MWH Model and other demand-generating interventions including SMGL, women in both arms still perceived barriers to facility-based delivery, including distance and transportation, and costs associated with delivery, consistent with baseline evaluation findings.[19] Health facilities in these rural districts serve catchment areas of 5,000 to 11,000 individuals.[44] Because

For peer review only - http://bmjopen.bmj.com/site/about/guidelines.xhtml

of the geography and low population density in the rural areas, health facilities are spread out and those fully equipped to manage obstetric complications are even fewer. In the near future, human resource and cost requirements make it unlikely that the GRZ could feasibly bring health services, particularly basic emergency obstetric services, within 5km of all populations (a WHO core health service access indicator for health systems).[45] Government officials in Zambia and other countries with similar access issues should consider MWHs an alternative option to make health facilities physically accessible to the most rural populations. Though MWHs address the distance barrier, they alone do not address the transportation barriers that persist here and that have been widely reported in areas similar to rural Zambia.[4,46,47] Policy makers should apply systems thinking to adopt financing policies that will provide benefits to rural residents, and expand monitoring for urban/rural inequities.[48,49]

Not surprisingly, MWH availability was more heavily discussed amongst participants in the intervention arm where the Core MWH Model was implemented, though the theme also emerged amongst control participants, indicating an overall awareness of MWH in the study areas. MWHs help women to overcome the distance and transportation barriers, allowing them to better plan and prepare to be close to health facilities when nearing delivery. Additionally, the proximity of MWHs to health facilities theoretically allows women to be easily attended by staff during the final weeks of pregnancy and when labor begins, increasing the likelihood for a safe delivery, one of the three perceived major drivers affecting women's decision to deliver at health facilities. A quantitative study in Zambia showed that health facilities with any MWH or waiting space, regardless of quality, had a slightly higher proportion of deliveries compared to those that had no waiting space, however, women were even more likely to deliver at health facilities with higher quality MWHs versus those with low-quality MWHs.[14]

Women also explained that penalties for home deliveries influenced their decision making, saying they would deliver at a health facility to avoid paying penalties to the health facility or to

For peer review only - http://bmjopen.bmj.com/site/about/guidelines.xhtml

traditional leadership, consistent with findings from two previous studies in rural Zambia.[31,50] However, some women noted they did not witness any penalties being enforced in their communities. Similarly, when rural Zambian women were previously asked to enumerate all costs associated with delivery, penalties among women delivering at home were insignificant compared to all other delivery costs.[51] Penalties may exist only as a rumor within these populations, or are levied in limited geographic areas. Further informal queries revealed that levying penalties for home delivery is not a law or official GRZ regulation, and that the government discourages such practices. Regardless, the belief that penalties for home delivery exist is a motivator for women to deliver at a facility.

The concern over the cost of facility-based delivery also persisted. Zambian women are required by facility staff to provide delivery supplies, and they perceive the need to provide new items, particularly baby clothes, when delivering at a health facility to avoid embarrassment or being shouted at by staff.[43] A study in rural Zambia also found that women who had saved more before coming to a health facility for delivery were less likely to report feeling disrespected. [52] Previous research in these areas of rural Zambia found delivery supplies, such as clamps and cotton wool, represent a small percentage of the overall delivery expenditure, while baby clothes and a baby blanket were the drivers of expenses.[51] Consistent with our qualitative findings, this quantitative study also showed that expenditure differed by delivery location, with women delivering at facilities reporting higher expenditures than those delivering at home.[51]

Women perceived MWHs may reduce access barriers due to distance, facilitating increased use of delivery and postnatal health services among remote-living women corroborating the quantitative findings from this study.[53] However, perceived challenges with transportation and costs associated with delivering at a facility or utilizing a MWH pose a potential barrier to their use, consistent with other studies.[43,54] Interventions that effectively address these challenges should continue to be explored and implemented.

For peer review only - http://bmjopen.bmj.com/site/about/guidelines.xhtml

Study Strengths

This study has several strengths. First, it provides insight on how MWH characteristics (i.e., Core MWH Model versus existing MWHs) may influence perceptions about barriers to facility-based delivery. Second, because we did not know the range of variation to expect in our population, our method of using a randomly selected, representative sample of the population assured the best opportunity for diversity in perceptions.[55,56] Lastly, the rural population sampled for this study is a hard-to-reach population that likely faces the greatest barriers to facility delivery due to their low socio-economic status, poor road infrastructure, limited transportation options, and low densities of facilities and skilled birth attendants. Using the qualitative data collected through this strategy, we were able to more deeply understand what influences these women's delivery location across seven culturally diverse and geographically dispersed study districts, providing important information for policy makers and program implementers interested in serving these vulnerable populations.

Study Limitations

While this study has multiple strengths, there were also several limitations. First, our findings are subject to recall bias as we interviewed women who delivered up to 12 months prior to data collection. However, we primarily asked about what influences the location of delivery for "women in this community," which asks about social norm and is less subject to recall bias than personal experiences and decision-making.[55] Second, it is likely that the presence of this study resulted in increased awareness of MWHs among control populations, as shown by the main quantitative study findings published separately.[22] We conducted multiple rounds of data collection for both the impact (baseline and endline) and implementation studies where community members and health system staff were asked about MWHs. Third, we did not disaggregate by MWH users and non-users or by location of MWH stay or delivery location. All these personal experiences likely influenced women's responses. We sought to understand how community-level perceptions changed over time, not just individual-level

For peer review only - http://bmjopen.bmj.com/site/about/guidelines.xhtml

decision-making. A different analysis could be conducted to understand responses from women based on their MWH use or delivery location. Lastly, our intervention was implemented in a specific policy environment, and in SMGL-supported districts, limiting generalizability beyond the study districts.

CONCLUSION

High quality MWHs can help to address some of the well-known perceived barriers to facility-based delivery; however other obstacles persist. While MWHs may be useful to address the distance barriers, no single intervention is likely to address all the barriers experienced by remote, rural, and poor populations. The quantitative and qualitative results from this study suggest MWHs could be considered in a broader systems approach to improving access in remote areas by policy holders and implementers.

Contributions

Co-principal investigators NAS and JRL acquired funding and conceived the research question and designed the trial. Along with NAS and JRL, co-investigators PCR, DHH, TV, MLMK, EAM, and GB contributed to conceptualization of the study, development of the research questions, and design of the trial, sampling methodology, and instruments. NAS, JLK, RMF, TV, and TN oversaw investigation, research methodology, and data acquisition. JLK, RMF, TN, MB, and VRS were responsible for data curation. RMF conducted the data analysis. TN, GB, and GM participated in study coordination provincially and nationally. MB, VRS, and GM implemented the interventions and oversaw project administration. RMF, JLK, KJK, TV, and NAS wrote the original draft of this manuscript. All authors reviewed and edited the manuscript and approved the final version.

Funding statement

For peer review only - http://bmjopen.bmj.com/site/about/guidelines.xhtml

This program was developed and implemented in collaboration with Merck for Mothers, Merck's 10-year, $500 million initiative to help create a world where no woman dies giving life. Merck for Mothers is known as MSD for Mothers outside the United States and Canada (MRK 1846-06500.COL). The development of this article was also supported in part by the Bill & Melinda Gates Foundation (OPP1130334) https://www.gatesfoundation.org/How-We-Work/Quick-Links/Grants-Database/Grants/2015/06/OPP1130334 and The ELMA Foundation (ELMA-15-F0010) http://www.elmaphilanthropies.org/the-elma-foundation/. The funders had no role in study design, data collection and analysis, decision to publish, or preparation of the manuscript. The content is solely the responsibility of the authors and does not necessarily reflect positions or policies of Merck, the Bill & Melinda Gates Foundation, or The ELMA Foundation. Funding for Online Open publication supported by Chronos Support through the Bill & Melinda Gates Foundation.

Declaration of Interest

All authors report their institutions received grants directly or indirectly from the Bill & Melinda Gates Foundation, MSD for Mothers, and The ELMA Foundation to conduct this study.

Acknowledgments

We appreciate the efforts, commitment, and contributions of the project staff, administrative support staff, the data collection team, and interviewers. From the Boston University/Right to Care team, we are thankful for the contributions of Denson Chongwe, David Kalaba, Deophine Bwalya, Parker Chastain, Allison Juntunen, Kathleen McGlasson, Elizabeth Ragan, Carey Howard, and Jason Park. From the Africare/University of Michigan team, we appreciate the efforts of Anne Naggyi, Isaac Sakala, Jessy Mtenje, Elizabeth Simwawa, Tenford Phiri, Lupiya Chilambwe, Jameson Kaunda, Nchimunya Chiboola and Nancy Lockhart. We are thankful to the team of translators and transcribers, and the two qualitative

For peer review only - http://bmjopen.bmj.com/site/about/guidelines.xhtml

data coders Ingrid Olson and Jordan Musante for their hard work. We acknowledge the ongoing support of the Zambian Ministry of Health, the Provincial Health Offices, the District Health Offices; we thank Mary Nambao for her support and advice. We thank the Chiefs who welcomed us into their communities. This study could not have happened without the tireless efforts of the health facility staff and community-based volunteers who were champions of the intervention, advisors to project staff, and community guides to data collection teams. Finally, we thank the women, their infants, and their families for welcoming data collection teams into their homes and participating in this study. This research was conducted with funding from MSD for Mothers, the Bill & Melinda Gates Foundation, and The ELMA Foundation. The findings and conclusions are those of the authors and do not necessarily reflect the policies or positions of the funders.

Data Sharing Statement

De-identified data for this analysis will be made available through OpenBU. The OpenBU repository has data access policies and procedures consistent with NIH data sharing policies.

References

1 World Health Organization. WHO recommendations on health promotion interventions for maternal and newborn health. Geneva, Switzerland: 2015. doi:10.1017/CBO9781107415324.004

2 Wong KLM, Benova L, Campbell OMR. A look back on how far to walk: Systematic review and meta-analysis of physical access to skilled care for childbirth in Sub-Saharan Africa. *PLoS One* 2017;**12**:e0184432. doi:10.1371/journal.pone.0184432

3 Sacks E, Vail D, Austin-Evelyn K, *et al.* Factors influencing modes of transport and travel time for obstetric care: a mixed methods study in Zambia and Uganda. *Health Policy Plan* 2016;**31**:293–301. doi:10.1093/heapol/czv057

4 Kyei-Nimakoh M, Carolan-Olah M, McCann T V. Access barriers to obstetric care at health facilities in sub-Saharan Africa-a systematic review. *Syst Rev* 2017;**6**:1–16. doi:10.1186/s13643-017-0503-x

5 Sialubanje C, Massar K, Hamer DH, *et al.* Understanding the psychosocial and environmental factors and barriers affecting utilization of maternal healthcare services in Kalomo, Zambia: A

For peer review only - http://bmjopen.bmj.com/site/about/guidelines.xhtml

qualitative study. *Health Educ Res* 2014;**29**:521–32. doi:10.1093/her/cyu011

6 Stekelenburg J, Kyanamina S, Mukelabai M, *et al.* Waiting too long: Low use of maternal health services in Kalabo, Zambia. *Trop Med Int Heal* 2004;**9**:390–8. doi:10.1111/j.1365-3156.2004.01202.x

7 Fogliati P, Straneo M, Mangi S, *et al.* A new use for an old tool: maternity waiting homes to improve equity in rural childbirth care. Results from a cross-sectional hospital and community survey in Tanzania. *Health Policy Plan* 2017;**32**:1354–60. doi:10.1093/heapol/czx100

8 Republic of Zambia Ministry of Health. National Health Strategic Plan 2011 - 2015. Lusaka, Zambia: 2010.

9 Republic of Zambia Ministry of Health. National Health Strategic Plan 2006-2010. Lusaka, Zambia: 2005.

10 Masiye F, Chitah BM, McIntyre D. From targeted exemptions to user fee abolition in health care: Experience from rural Zambia. *Soc Sci Med* 2010;**71**:743–50. doi:10.1016/j.socscimed.2010.04.029

11 Ministry of Health (MOH) Zambia, Ministry of Community Development Mother and Child Health (MCDMCH) Zambia. Roadmap for Accelerating Reduction of Maternal , Newborn and Child Mortality, 2013-2016. 2013.

12 Morrissey Conlon C, Serbanescu F, Marum L, *et al.* Saving Mothers , Giving Life: It takes a system to save a mother. *Glob Heal Sci Pract* 2019;**7**:S6–26. doi:https://doi.org/10.9745/GHSP-D-18-00427

13 Serbanescu F, Clark TA, Goodwin MM, *et al.* Impact of the SMGL approach on decreasing maternal and perinatal deaths in Uganda and Zambia. *Glob Heal Sci Pract* 2019;**7**:S29–47. doi:https://doi.org/10.9745/GHSP-D-18-00428

14 Henry EG, Semrau K, Hamer DH, *et al.* The influence of quality maternity waiting homes on utilization of facilities for delivery in rural Zambia. *Reprod Health* 2017;**14**:68. doi:10.1186/s12978-017-0328-z

15 Lori JR, Wadsworth AC, Munro ML, *et al.* Promoting access: The use of maternity waiting homes to achieve safe motherhood. *Midwifery* 2013;**29**:1095–102. doi:10.1016/j.midw.2013.07.020

16 Stekelenburg J, Van Lonkhuijzen L, Spaans W, *et al.* Maternity waiting homes in rural districts in Africa: A cornerstone of safe motherhood? *Curr Womens Health Rev* 2006;**2**:235–8. doi:10.2174/157340406778699914

17 Penn-Kekana L, Pereira S, Hussein J, *et al.* Understanding the implementation of maternity waiting homes in low- and middle-income countries: a qualitative thematic synthesis. *BMC Pregnancy Childbirth* 2017;**17**:269. doi:10.1186/s12884-017-1444-z

18 Gabrysch S, Cousens S, Cox J, *et al.* The influence of distance and level of care on delivery place in rural Zambia: a study of linked national data in a geographic information system. *PLoS Med* 2011;**8**. doi:10.1371/journal.pmed.1000394

19 Scott NA, Henry EG, Kaiser JL, *et al.* Factors affecting home delivery among women living in remote areas of rural Zambia : a cross-sectional, mixed-methods analysis. *Int J Womens Health*

For peer review only - http://bmjopen.bmj.com/site/about/guidelines.xhtml

2018;**10**:589–601.

20 Lori JR, Munro ML, Rominski S, *et al.* Maternity waiting homes and traditional midwives in rural Liberia. *Int J Gynecol Obstet* 2013;**123**:114–8. doi:10.1016/j.ijgo.2013.05.024

21 Perosky JE, Munro-Kramer ML, Lockhart N, *et al.* Maternity waiting homes as an intervention to increase facility delivery in rural Zambia. *Int J Gynecol Obstet* 2019;**146**:266–7. doi:10.1002/ijgo.12864

22 Scott NA, Kaiser JL, Ngoma T, *et al.* If we build it, will they come? Results of a quasi-experimental trial assessing the impact of maternity waiting homes on facility-based childbirth and maternity care in Zambia. *BMJ Glob Heal* 2021;**6**. doi:10.1136/bmjgh-2021-006385

23 Scott NA, Vian T, Kaiser JL, *et al.* Listening to the community: Using formative research to strengthen maternity waiting homes in Zambia. *PLoS One* 2018;**13**:e0194535. doi:https://doi.org/10.1371/journal.pone.0194535

24 Lori JR, Munro-Kramer ML, Mdluli EA, *et al.* Developing a community driven sustainable model of maternity waiting homes for rural Zambia. *Midwifery* 2016;**41**:89–95. doi:10.1016/j.midw.2016.08.005

25 Scott NA, Kaiser JL, Vian T, *et al.* Impact of maternity waiting homes on facility delivery among remote households in Zambia: protocol for a quasiexperimental, mixed-methods study. *BMJ Open* 2018;**8**. doi:10.1136/bmjopen-2018-022224

26 Lori JR, Munro-Kramer ML, Liu H, *et al.* Increasing facility delivery through maternity waiting homes for women living far from a health facility in rural Zambia: a quasi-experimental study. *BJOG An Int J Obstet Gynaecol* Published Online First: 2021. doi:10.1111/1471-0528.16755

27 Henry EG, Ngoma T, Kaiser JL, *et al.* Evaluating implementation effectiveness and sustainability of a maternity waiting homes intervention to improve access to safe delivery in rural Zambia: a mixed-methods protocol. *BMC Health Serv Res* 2020;**20**:191. doi:10.1186/s12913-020-4989-x

28 Bonawitz R, McGlasson KL, Kaiser JL, *et al.* Quality and utilization patterns of maternity waiting homes at referral facilities in rural Zambia: A mixed-methods multiple case analysis of intervention and standard of care sites. *PLoS One* 2019;**14**:1–18. doi:10.1371/journal.pone.0225523

29 Central Statistical Office (CSO) Zambia. Zambia 2010 Census of Population and Housing National Analytical Report. 2012.

30 Central Statistical Office Zambia. 2015 Living Conditions Monitoring Survey Report. 2016. http://www.zamstats.gov.zm/report/Lcms/2006-2010 LCMS Report Final Output.pdf

31 Greeson D, Sacks E, Masvawure TB, *et al.* Local adaptations to a global health initiative: penalties for home births in Zambia. *Health Policy Plan* 2016;:czw060. doi:10.1093/heapol/czw060

[dataset] 32 Scott NA, Kaiser JL, Ngoma T, *et al.* Data from: If we build it, will they come? Results of a quasi-experimental study assessing the impact of maternity waiting homes on facility-based childbirth and maternity care in Zambia. *OpenBU, November 23, 2020.* https://hdl.handle.net/2144/43100

[dataset] 33 Fong RM, Kaiser JL, Ngoma T, *et al.* Data from: Barriers and facilitators to facility-based

For peer review only

For peer review only - http://bmjopen.bmj.com/site/about/guidelines.xhtml

delivery in rural Zambia: A qualitative study of women's perceptions after implementation of an improved Maternity Waiting Homes intervention. *OpenBU, November 16, 2019.* doi:[awaiting institutional approval]

34 Dobility INC. SurveyCTO [Technology for digical data collection]. 2017.

35 Gale NK, Heath G, Cameron E, *et al.* Using the framework method for the analysis of qualitative data in multi-disciplinary health research. *BMC Med Res Methodol* 2013;**13**:117. doi:10.1186/1471-2288-13-117

36 Ritchie J, Lewis J. *Qualitative research practice: a guide for social science students and researchers*. London: 2003. doi:March 10, 2016

37 Becker MH. The Health Belief Model and Personal Health Behavior. *Health Educ Monogr* 1974;**2**:324–508.http://dx.doi.org/10.1177/109019817400200407

38 O'Brien BC, Harris IB, Beckman TJ, *et al.* Standards for reporting qualitative research: a synthesis of recommendations. *Acad Med* 2014;**89**:1245–51. doi:10.1097/ACM.0000000000000388

39 Vian T, Fong RM, Kaiser JL, *et al.* Using Open Public Meetings and Elections to Promote Inward Transparency and Accountability: Lessons From Zambia. *Int J Heal Policy Manag* 2020;:1–13. doi:10.34172/ijhpm.2020.84

40 Kaiser JL, Fong RM, Ngoma T, *et al.* The effects of maternity waiting homes on the health workforce and maternal health service delivery in rural Zambia: A qualitative analysis. *Hum Resour Health* 2019;**17**. doi:10.1186/s12960-019-0436-7

41 Fontanet CP, Fong RM, Kaiser JL, *et al.* A qualitative exploration of community ownership of a maternity waiting home model in rural zambia. *Glob Heal Sci Pract* 2020;**8**:344–57. doi:10.9745/GHSP-D-20-00136

42 Sakanga VI, Chastain P, McGlasson KL, *et al.* Building financial management capacity for community ownership of development initiatives in rural Zambia. *Int J Health Plann Manage* 2019;**34**. doi:10.1002/hpm.2810

43 Sialubanje C, Massar K, G van der Pijl MS, *et al.* Improving access to skilled facility-based delivery services: Women's beliefs on facilitators and barriers to the utilisation of maternity waiting homes in rural Zambia. *Reprod Health* 2015;**12**. doi:10.1186/s12978-015-0051-6

44 Republic of Zambia Ministry of Health. The 2012 List of Health Facilities in Zambia. 2013. http://www.moh.gov.zm/docs/facilities.pdf (accessed 31 May 2018).

45 World Health Organization. Global Reference List of 100 Core Health Indicators. Geneva, Switzerland: 2015. apps.who.int/iris/bitstream/10665/173589/1/WHO_HIS_HSI_2015.3_eng.pdf%0Ahttp://ci.nii.ac.jp/naid/40020358184/

46 Moyer CA, Mustafa A. Drivers and deterrents of facility delivery in sub-Saharan Africa: a systematic review. *Reprod Health* 2013;**10**:1–14. doi:10.1186/1742-4755-10-40

47 Bohren MA, Hunter EC, Munthe-Kaas HM, *et al.* Facilitators and barriers to facility-based delivery in low- and middle-income countries: a qualitative evidence synthesis. *Reprod Health* 2014;**11**:71. doi:10.1186/1742-4755-11-71

For peer review only

For peer review only - http://bmjopen.bmj.com/site/about/guidelines.xhtml

48 Zhang X, Bloom G, Xu X, *et al.* Advancing the application of systems thinking in health: Managing rural China health system development in complex and dynamic contexts. *Health Qual Life Outcomes* 2014;**12**:1–9. doi:10.1186/1478-4505-12-44_old

49 Hosseinpoor AR, Bergen N, Koller T, *et al.* Equity-Oriented Monitoring in the Context of Universal Health Coverage. *PLoS Med* 2014;**11**. doi:10.1371/journal.pmed.1001727

50 Chibuye PS, Bazant ES, Wallon M, *et al.* Experiences with and expectations of maternity waiting homes in Luapula Province, Zambia: a mixed–methods, cross-sectional study with women, community groups and stakeholders. *BMC Pregnancy Childbirth* 2018;**18**:42. doi:10.1186/s12884-017-1649-1

51 Kaiser JL, Mcglasson KL, Rockers PC, *et al.* Out-of-pocket expenditure for home and facility- based delivery among rural women in Zambia : a mixed-methods, cross-sectional study. *Int J Womens Health* 2019;**11**:411–30. doi:10.2147/IJWH.S214081

52 Chiu C, Scott NA, Kaiser JL, *et al.* Household saving during pregnancy and facility delivery in Zambia: a cross-sectional study. *Health Policy Plan* 2019;:1–8. doi:10.1093/heapol/czz005

53 Lori J, Munro-Kramer M, Liu H, *et al.* Increasing facility delivery through maternity waiting homes for women living far from a health facility in rural Zambia: a quasi-experimental study. *Br J Obstet Gynaecol* Published Online First: 2021. doi:10.1111/1471

54 Gaym A, Pearson L, Soe KWW. Maternity waiting homes in Ethiopia--three decades experience. *Ethiop Med J* 2012;**50**:209–19.

55 Miles MB, Huberman AM. *Qualitative Data Analysis: An Expanded Sourcebook, 2nd Edition*. 2nd ed. Thousand Oaks, California: : SAGE Publications, Inc 1994.

56 Patton M. *Qualitative Research and Evaluation methods, 3rd Edition*. 3rd ed. Thousand Oaks, California: : SAGE Publications 2002.

For peer review only - http://bmjopen.bmj.com/site/about/guidelines.xhtml

SURVEY ID [ ]

Instrument ID:

The MAHMAZ Project
Endline Impact Evaluation – In Depth Interview Instrument **ENGLISH**

Target Audience:
Women who have delivered a child in the last 12 months, who are ≥ 15 years of age, and who live within the study facility catchment areas

Was verbal informed consent (or assent/consent if age 15-17) obtained for this interview during the process of informed consent for the household survey?

☐ **YES**

☐ **NO – STOP!** Thank the participant for their time. Do NOT proceed with the interview.

Step 1: *Read the following statement. Please repeat the statement translated into the local language based on primary languages.*

"Thank you for agreeing to participate in this interview. My name is ______. I will be asking you questions during this interview and recording your responses on this machine."

"We want to understand in greater detail your and your community's views on pregnancy and delivery and your perspectives on mothers' shelters. Please feel free to tell us only what you feel comfortable sharing. There are no right or wrong answers, so please be honest and help us to understand what is true for you and your community. Are you ready to begin?"

Step 2: *Proceed to the interview guide. Please probe to obtain as in-depth and specific information you can.*

.

Interviewer Name: ____________________

1. **Interview Date:**

				**2**	**0**	**1**	**8**
MM		DD		YY			

2. **Time Start:**

		**:**		
**H**	**H**		**M**	**M**

3. **Time Finish:**

		**:**		
**H**	**H**		**M**	**M**

THIS FORM MUST BE TURNED INTO YOUR FIELD COORDINATOR FOR EVERY IDI YOU CONDUCT.

For peer review only

For peer review only - http://bmjopen.bmj.com/site/about/guidelines.xhtml

SURVEY ID

READ THE DATE, UNIQUE ID, and START TIME ON THE RECORDING BEFORE YOU BEGIN THE INTERVIEW.

"Thank you for agreeing to speak with me today. I'm going to ask you some questions about people in your community and some questions about your own experience."

Theme 1: Delivery practices and location:

1a. Where do most pregnant women in your community deliver their babies? (e.g., home, health center, hospital) **Why do they do so?**

1b. What **factors** do people in your community mostly consider when deciding where to deliver?

Probe for (ask each separately after the woman has initially responded. We want more than just yes/no answers, ask for detail!):

- Do people consider **safety**? How?
- Do people consider **cultural preferences**? How?
- Do people consider **comfort**? How?
- Do people consider **cleanliness**? How?
- Do people consider **the opinions of others** – health worker, SMAG, spouses, mother-in-law, etc. How?
- Do people consider **distance**? How?
- Do people consider **transport**? How?
- Do people consider **cost of delivering at a facility**? How?
- Do people consider the **health facility requirements** for delivery? How?
- Do people consider the presence of a **Mothers' Shelter**? How?
- Do people consider the **quality of care provided** by health facility staff? How?
- Do people consider **local rules/laws**? How?

1c. Please tell me a story of **your delivery journey**, of all the places you went to or intended to go.

Rephrase if needed: What happened on the days leading up to delivery, the day you delivered, and in the days after?

For peer review only - http://bmjopen.bmj.com/site/about/guidelines.xhtml

SURVEY ID

1d. Tell me what factors were involved in the decision of where you delivered your last baby? What was the most important of these?

Probe for (ask each separately after the woman has initially responded!):

- Do people consider **safety**? How?
- Do people consider **cultural preferences**? How?
- Do people consider **comfort**? How?
- Do people consider **cleanliness**? How?
- Do people consider **the opinions of others** – health worker, SMAG, spouses, mother-in-law, etc. How?
- Do people consider **distance**? How?
- Do people consider **transport**? How?
- Do people consider **cost of delivering at a facility**? How?
- Do people consider the **health facility requirements** for delivery? How?
- Do people consider the presence of a **Mothers' Shelter**? How?
- Do people consider the **quality of care provided** by health facility staff? How?
- Do people consider **local rules/laws**? How?

1e. Who helped you decide where to deliver your last baby? Of those, who was most influential for you when you were deciding where to deliver your baby? **Why?**

Probe for: *yourself, spouse, mother-in-law, aunties, self, SMAGs, health worker, etc.*

1f. Are there penalties for delivering at home? What are the penalties (monetary/non-monetary)? How much/how many? Who enforces them?

Theme 2: Mother's Shelters

2a. In general, what do people think of mothers' shelters in your community?

2a1. Do people in your community use mothers' shelters? **Why or why not?**

2b. Where do people in your community hear about mothers' shelters?

Probe with: *From men/spouses, mother-in-law, other mothers elder, family, chiefs, ANC, SMAG, health worker, radio etc.*

2c. In general, what do people in your community think of the quality of mother shelters?

For peer review only - http://bmjopen.bmj.com/site/about/guidelines.xhtml

SURVEY ID

Probe with:

- What do people say about their **comfort**?
- What do people say about their **safety**? **Why**?
- What do people say about how they are **managed**?
- What do people say about the **linkages with the health facility**?
- What do people say about the **classes offered** there?

2d. If you have stayed at the mothers' shelter, please tell me about **your overall experience**. Would you stay again? Why or why not?

2e. Please tell me a story about a **positive experience** you have had at or have heard about the mothers' shelter.

2f. Please tell me a story about a **negative experience** you have had at or have heard about the mothers' shelter.

2g. What makes it **hard to use** the mothers' shelter in your community?

2h. What makes it **easy to use** the mothers' shelter in your community?

2i. Who **owns** the mothers' shelter? Please explain.

2j. How can the mothers' shelter in your community be **improved**?

Theme 3: Preparedness and Costs

3a. What did you do to plan for your delivery?
Probe for: *costs, transport, supplies, baby clothes, etc.*

3b. About how far along were you in your pregnancy when you began to plan for delivery?

For peer review only - http://bmjopen.bmj.com/site/about/guidelines.xhtml

For peer review only

SURVEY ID

3c. Who helped you plan for delivery? What did they do to help?
Probe for: *health provider at ANC, SMAGs, mother-in-law, spouse, friends, etc.*

3d. You delivered your most recent child at (Health facility/hospital/home/other). **Tell me what it cost you to deliver your most recent baby?** Please be specific.

Probe for: *preparations, supplies, gifts, assistance fee, transport*

3e. Would the costs have been different if you delivered at (health facility/hospital/home/other)? If yes, how so?

- What is the largest cost associated with delivering a baby in the facility?
  - On average, how much does this cost?

3f. When you planned for delivery did you **save money**? If so, how did you save money?

3g. What were the main **challenges** associated with saving money for delivery?

3h. Do you think you **saved enough**? Please explain.

3i. Do other people in the community contribute to a woman's delivery savings?
Probe for: Money or non-monetary items

3j. Did you or someone you know take part in a **savings group**? Please tell me about that experience.

"I have finished with this in-depth interview. Is there anything you would like to add before we end?"

Thank you so much for your time.

For peer review only - http://bmjopen.bmj.com/site/about/guidelines.xhtml

Standards for Reporting Qualitative Research (SRQR)

O'Brien B.C., Harris, I.B., Beckman, T.J., Reed, D.A., & Cook, D.A. (2014). Standards for reporting qualitative research: a synthesis of recommendations. *Academic Medicine, 89(9)*, 1245-1251.

No. Topic	Item	Page number
**Title and abstract**		
S1 Title	Concise description of the nature and topic of the study identifying the study as qualitative or indicating the approach (e.g., ethnography, grounded theory) or data collection methods (e.g., interview, focus group) is recommended	Page 1
S2 Abstract	Summary of key elements of the study using the abstract format of the intended publication; typically includes objective, methods, results, and conclusions	Page 3
**Introduction**		
S3 Problem formulation	Description and significance of the problem/phenomenon studied; review of relevant theory and empirical work; problem statement	Page 5-6, page 7 lines 1-3
S4 Purpose or research question	Purpose of the study and specific objectives or questions	Page 7, lines 3-7
**Methods**		
S5 Qualitative approach and research paradigm	Qualitative approach (e.g., ethnography, grounded theory, case study, phenomenology, narrative research) and guiding theory if appropriate; identifying the research paradigm (e.g., positivist, constructivist/interpretivist) is also recommended	Page 9, lines 7-17<br><br>Page 10, lines 13-24
S6 Researcher characteristics and reflexivity	Researchers' characteristics that may influence the research, including personal attributes, qualifications/experience, relationship with participants, assumptions, or presuppositions; potential or actual interaction between researchers' characteristics and the research questions, approach, methods, results, or transferability	Page 10, lines 2-3<br><br>Page 10, lines 12-13
S7 Context	Setting/site and salient contextual factors; rationale[a]	Page 7, lines 11-23
S8 Sampling strategy	How and why research participants,	Page 9, lines 4-9

For peer review only

	documents, or events were selected; criteria for deciding when no further sampling was necessary (e.g., sampling saturation); rationale[a]	and lines 19-24
S9 Ethical issues pertaining to human subjects	Documentation of approval by an appropriate ethics review board and participant consent, or explanation for lack thereof; other confidentiality and data security issues	Page 11, lines 18-24
S10 Data collection methods	Types of data collected; details of data collection procedures including (as appropriate) start and stop dates of data collection and analysis, iterative process, triangulation of sources/methods, and modification of procedures in response to evolving study findings; rationale[a]	Page 9, lines 4-9 and lines 15-17<br><br>Page 10, lines 2-9<br><br>Page 11, lines 1-2
S11 Data collection instruments and technologies	Description of instruments (e.g., interview guides, questionnaires) and devices (e.g., audio recorders) used for data collection; if/how the instrument(s) changed over the course of the study	Page 9, lines 5-15<br><br>Page 10, line 3-8
S12 Units of study	Number and relevant characteristics of participants, documents, or events included in the study; level of participation (could be reported in results)	Page 9, lines 19-24<br><br>Page 12, lines 3-7<br><br>Table 1
S13 Data processing	Methods for processing data prior to and during analysis, including transcription, data entry, data management and security, verification of data integrity, data coding, and anonymization/deidentification of excerpts	Page 10, lines 11-24<br><br>Page 11, 1-9
S14 Data analysis	Process by which inferences, themes, etc., were identified and developed, including researchers involved in data analysis; usually references a specific paradigm or approach; rationale[a]	Page 10, lines 13-24<br><br>Page 11, lines 1-2
S15 Techniques to enhance trustworthiness	Techniques to enhance trustworthiness and credibility of data analysis (e.g., member checking, audit trail, triangulation); rationale[a]	Page 10, lines 11-13 and lines 15-19<br><br>Page 11, lines 1-2
**Results/Findings**		
S16 Synthesis and interpretation	Main findings (e.g., interpretations, inferences, and themes); might include development of a theory or model, or integration with prior research or theory	Page 13-14, Page 17-18, Page 19 lines 1-7
S17 Links to empirical data	Evidence (e.g., quotes, field notes, text excerpts, photographs) to substantiate analytic findings	Page 15-16 (Table 3)
**Discussion**		
S18 Integration with prior	Short summary of main findings; explanation	Page 19 lines 10-

For peer review only

work, implications, transferability, and contribution(s) to the field	of how findings and conclusions connect to, support, elaborate on, or challenge conclusions of earlier scholarship; discussion of scope of application/generalizability; identification of unique contribution(s) to scholarship in a discipline or field	24, Page 20-21
S19 Limitations	Trustworthiness and limitations of findings	Page 22, lines 14-24<br><br>Page 23, lines 1-3
**Other**		
S20 Conflicts of interest	Potential sources of influence or perceived influence on study conduct and conclusions; how these were managed	Page 24, lines 10-12
S21 Funding	Sources of funding and other support; role of funders in data collection, interpretation, and reporting	Page 23, lines 22-24<br><br>Page 24, lines 1-8

[a]The rationale should briefly discuss the justification for choosing that theory, approach, method, or technique rather than other options available, the assumptions and limitations implicit in those choices, and how those choices influence study conclusions and transferability. As appropriate, the rationale for several items might be discussed together.

For peer review only
